# Universal Spatial Correlation Functions for Describing and Reconstructing Soil Microstructure

**DOI:** 10.1371/journal.pone.0126515

**Published:** 2015-05-26

**Authors:** Marina V. Karsanina, Kirill M. Gerke, Elena B. Skvortsova, Dirk Mallants

**Affiliations:** 1 Institute of Geospheres Dynamics of the Russian Academy of Sciences, Moscow, Russia; 2 AIR Technology, Moscow, Russia; 3 CSIRO Land and Water, Adelaide, South Australia, Australia; 4 Dokuchaev Soil Science Institute of Russian Academy of Sciences, Moscow, Russia; University of Texas at San Antonio, UNITED STATES

## Abstract

Structural features of porous materials such as soil define the majority of its physical properties, including water infiltration and redistribution, multi-phase flow (e.g. simultaneous water/air flow, or gas exchange between biologically active soil root zone and atmosphere) and solute transport. To characterize soil microstructure, conventional soil science uses such metrics as pore size and pore-size distributions and thin section-derived morphological indicators. However, these descriptors provide only limited amount of information about the complex arrangement of soil structure and have limited capability to reconstruct structural features or predict physical properties. We introduce three different spatial correlation functions as a comprehensive tool to characterize soil microstructure: 1) two-point probability functions, 2) linear functions, and 3) two-point cluster functions. This novel approach was tested on thin-sections (2.21×2.21 cm^2^) representing eight soils with different pore space configurations. The two-point probability and linear correlation functions were subsequently used as a part of simulated annealing optimization procedures to reconstruct soil structure. Comparison of original and reconstructed images was based on morphological characteristics, cluster correlation functions, total number of pores and pore-size distribution. Results showed excellent agreement for soils with isolated pores, but relatively poor correspondence for soils exhibiting dual-porosity features (i.e. superposition of pores and micro-cracks). Insufficient information content in the correlation function sets used for reconstruction may have contributed to the observed discrepancies. Improved reconstructions may be obtained by adding cluster and other correlation functions into reconstruction sets. Correlation functions and the associated stochastic reconstruction algorithms introduced here are universally applicable in soil science, such as for soil classification, pore-scale modelling of soil properties, soil degradation monitoring, and description of spatial dynamics of soil microbial activity.

## Introduction

Soil microstructure, i.e. the spatial arrangement of mineral, organic, air and water, and other phases at the sub-Darcian scale, defines all local (i.e. at the scale of measurement) and effective (i.e. describing flow processes in an upscaled homogeneous medium, also referred-to as Darcy scale) soil properties. For example, pore structure and wettability properties of pore walls govern saturated hydraulic conductivity, capillary properties (water retention characteristic), and relative permeabilities for unsaturated water and gas flow. Pore-scale modeling approaches [1] have implemented these interdependencies [2–3] and have proven to be a valuable approach to predict porous media flow properties based on microscopic pore structure information [4–7]. In addition to filtration properties, structure defines such soil properties as molecular diffusion [8–10], mechanical properties [11–12], electrical resistivity [13–14], heat transfer and evaporation [15–17], and hydrodynamic dispersion [18–20]. These physical properties combined govern geochemical reactions and weathering [21–22], transport of solutes, nutrients, viruses or colloids [23–26], and affect living conditions of microorganisms [27–28]. In turn, such processes and the soil’s response to variable boundary conditions (e.g., precipitation, transpiration) will impact soil fertility and degradation [29], bio-clogging [30], irrigation and tillage management [31], and soil water storage under variable and changing climate [32]. In addition, given the importance of soil microstructure in defining other soil properties, its quantification should be incorporated in any characterization of basic soil properties. Throughout this paper, all references to the term ‘structure’ refer to microstructure.

Conventional methods to study soil structure include soil thin sections; more recently Scanning Electron Microscopy (SEM) and Back-scattering Imaging Microscopy (BSIM) have been used [8]. Both thin sections, SEM and BSIM usually result in 2D image information. X-ray micro-tomography (XMT) on the other hand, provides a very effective way of obtaining 3D images of soil structure with resolutions up to around 1 μm [33–34]. Although entire soil cores can be scanned with XMT, the larger the core size the lower the spatial resolution. Such 2/3D images have been used to study soil hydrological processes, structural changes and dynamic effects associated with biota influence and agricultural practices [35]. However, as soil structural properties are usually reported only as 2/3D visualizations, particle size/pore-size distributions, or fractal dimensions, a direct comparison of structural features across different soils is complicated in absence of a comprehensive soil structure descriptor.

Despite the broad recognition that soil structure and its relation to many physical properties and processes are invaluable in soil science, current research lacks a universal soil structure descriptor to characterize soil structure quantitatively and thus facilitate a universally applicable comparison for different soils. Such a descriptor should represent soil structure information in mathematical functions that allow solving the inverse problem, i.e. reconstruct soil structure from its descriptor function(s).

A variety of soil structure descriptors has been developed over the years, such as 1) particle size distributions plus organic matter content [36]; 2) pore-size distributions either from 3D pore structure images or water retention curves [37–38]; 3) fractal dimensions of soil pore space [4,20,39]; and 4) morphological measurements on thin-sections [29,40–41], and 5) Minkowski functionals [35,42].

While these sets of soil structure descriptors provide some first attempt to quantify structure, they are insufficient to reconstruct soil structure based on the available parameters. Indeed, even such simple structures as mono-disperse sphere packs can be extremely complex and possess different degrees of complexity [43–44]; for instance, similar sets of particles with defined grain sizes can be packed in many different ways. Furthermore, pore-sizes extracted from XMT images can be accurate only for pore sizes above resolution limit. Tortuosity and connectivity parameters inferred from water retention measurements or image analysis are highly uncertain due to non-uniqueness in the estimation of multiple parameters from often limited data [45–46]. As a result, the ability to predict capillary or multiphase flow properties for soils with important structural features is limited [47–48]. Reconstruction of even very simple synthetic structures using fractal dimension information is often of relatively poor quality [49]. For relatively simple structures typically observed in Berea or Fontainebleau sandstone, 2/3D morphological information used as input parameters to process-based reconstruction techniques has proven to yield acceptable reconstructions [50–52], but would fail in soils due to different genesis. Other novel methods such as Minkowski functional distributions [35,42] or local porosity/connectivity distributions [53] are potentially powerful methods to characterize soil structure and develop reconstructions. Their main drawback is that they are computationally expensive, which limits widespread application and could explain why applications to reconstruct structure have not yet been reported in the literature.

Another class of material structure description methods is based on spatial correlation functions (a detailed description is provided below). A particularly powerful reconstruction algorithm is one which employs a stochastic optimization approach based on simulated annealing [54], often referred to as the Yeong-Torquato technique [55]. Spatial correlation functions have been used in different disciplines to characterize and reconstruct numerous materials, including gels [56], sandstones [57–58], stars and galaxies [59], kerogen porosity in shales [60], concrete [61], filters [62–63], nanocomsposites [64–65], and soft matter such as food [66]. In our preliminary study [67] we applied spatial correlation functions (with averaged two-point probability computed only in orthogonal directions) for soil reconstructions based on the original Yeong-Torquato method which considers isotropic heterogeneous materials. On the basis of our recent modifications to the Yeong-Torquato method, which involves computing directional correlation functions [68], we demonstrate it is now possible to characterize complex soils with direction-dependent or anisotropic structures. The current paper builds on our previous work and is a first-ever demonstration of the predictive capacity of the novel directional spatial correlation functions [67–68] for soil structure quantification and reconstruction.

The objective of this paper is to apply novel direction-dependent spatial correlation functions to describe 2D soil binary structures (pore-solid images) and develop procedures for testing their usefulness by comparison of connectivity and other soil morphological descriptors based on original and reconstructed images.

## Materials and Methods

### Correlation functions

In our notion of correlation functions we mainly follow Torquato [2]. First, we introduce a binary indicator function *I* ^(*i*)^(***x***), which describes the affiliation between local points (pixels for 2D and voxels for 3D digitized images) of structure under study. For a two-phase system (e.g. solid-pore) the indicator function will take the following form in each location ***x*** in the two-dimensional Euclidian space ***R*** ^**2**^:

$$I^{\left(i\right)}\left(x\right)=\{\begin{array}{c} 1,\;x\in V_{i}\\ 0,\;x\in {\bar{V}}_{i} \end{array},$$ (1)

where ***V***_*i*_ ∊ ***R*** ^**2**^ is the region occupied by phase *i*, and ${\overset{-}{V}}_{i}\in R^{2}$ is the region occupied by the other phase. Next, a simple type of correlation function, i.e. the *n*-point probability function, *S*_*n*_, is defined which calculates the probability that *n* points lie in the same phase in the following manner:

$$S_{n}^{\left(i\right)}\left(x_{1},x_{2},\ldots ,x_{n}\right)=\langle I^{\left(i\right)}\left(x_{1}\right),I^{\left(i\right)}\left(x_{2}\right),\ldots ,I^{\left(i\right)}\left(x_{n})\right\rangle ,$$ (2)

where ***x*** is the coordinate vector and ⟨…⟩ denotes ensemble average**.** For a statistically homogeneous or stationary structure (i.e., the statistical descriptors of the geometrical arrangement do not depend on the position they are evaluated at), *n*-point probability functions depend only on the relative distances between points and not on their absolute coordinate values. This means:

$$S_{n}^{\left(i\right)}\left(x_{1},x_{2},\ldots ,x_{n}\right)=S_{n}^{\left(i\right)}\left(x_{12},x_{13},\ldots ,x_{1n}\right),$$ (3)

for all *n*≥1, where ***x***_*ij*_ = ***x***_*j*_ − ***x***_*i*_.

While *n*-point probability functions can theoretically be applied to soil thin-sections or XMT scans for describing structure, the computational cost is still prohibitive and a much simpler and hence efficient structure descriptor is required, especially in view of its use for structure reconstruction. As calculation of probability functions with *n*>3 involves numerous difficulties, applications so far have typically used a smaller number of points. Yeong and Torquato [55] argue that the complexity of calculations for *n*>2 is not offset by the gain in accuracy. Of the few applications of probability functions with *n* = 3 available in the literature [59], none relate to soil science.

**Fig 1:**
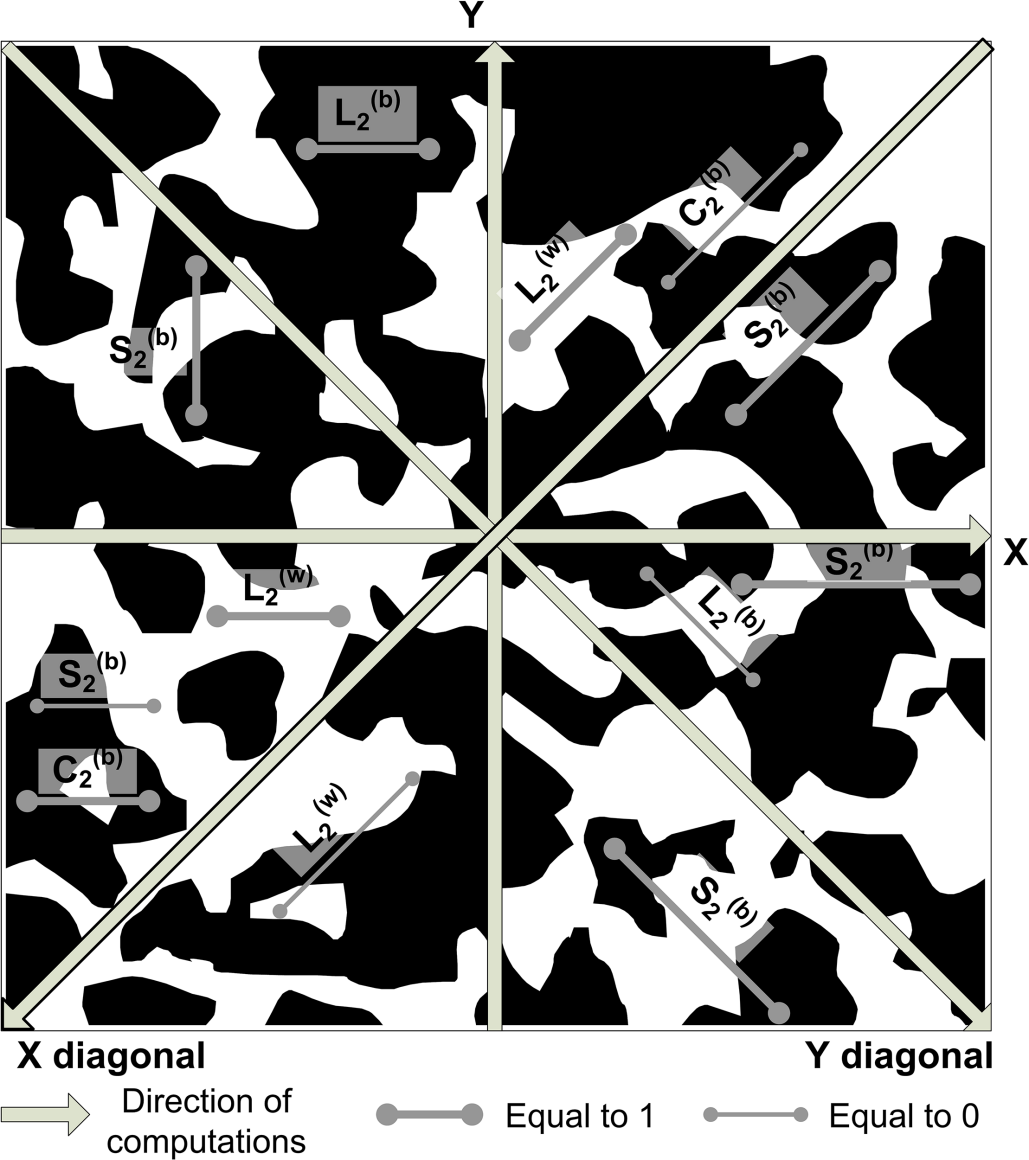
Schematic for correlation function computation in four principal directions (X, Y, X-diagonal and Y-diagonal) in a two-phase porous medium displaying pore (white areas) and solid (black areas) phase. Thick line segments represent examples providing correlation function’s local values of 1, while thin lines—0.

The two-point probability (*S*_*2*_) function is the lower-order version of *S*_*n*_ and represents the probability that two points separated by the vector displacement ***r***(*x*_1_,*x*_2_) between position vectors ***x***_1_ and ***x***_2_ lie in the same phase (pores or solids, represented by respectively white and black areas in Fig 1):

$$S_{2}^{\left(i\right)}\left(x_{1},x_{2}\right)=\langle I^{\left(i\right)}\left(x_{1}\right),I^{\left(i\right)}\left(x_{2})\right\rangle .$$ (4)

Analogous to Eq 3, one may write for statically homogeneous structures:

$$S_{2}^{\left(i\right)}\left(x_{1},x_{2}\right)=S_{2}^{\left(i\right)}\left(r\right),$$ (5)

where ***r*** = ***x***_12_ is the vector displacement between position vectors ***x***_1_ and ***x***_2_. For statistically homogeneous and isotropic structures the two-point probability functions will only depend on the scalar distance *r* between the points, or:

$$S_{2}^{\left(i\right)}\left(r\right)=S_{2}^{\left(i\right)}\left(r\right).$$ (6)

The first moment, *φ*_*i*_, of an *n*-point probability function, i.e., a one-point function:

$$S_{1}^{\left(i\right)}=S_{2}^{\left(i\right)}\left(0\right)=\langle I^{\left(i\right)}\left(x)\right\rangle =\varphi_{i}$$ (7)

is the probability of finding the point ***x*** to belong to phase *i*, or to a volumetric fraction (pore space or solid). For a binary, statistically homogeneous structure the relationship between two-point probability functions for each phase is given by (superscript *b* and *w* refer to respectively black and white areas in Fig 1):

$$S_{2}^{\left(b\right)}\left(r\right)=S_{2}^{\left(w\right)}\left(r\right)-2\varphi_{w}+1.$$ (8)

where *φ*_w_ is the probability of *x* being in the pore space. Using autocorrelation function notation (*φ*_b_ is the probability of *x* being in the solid space):

$$\chi \left(r\right)=S_{2}^{\left(w\right)}\left(r\right)-\varphi_{w}{}^{2}=S_{2}^{\left(b\right)}\left(r\right)-\varphi_{b}{}^{2}.$$ (9)

It is noted from Eqs 8 and 9 that two-point probability functions do not discriminate between binary phases. This means that one cannot improve statistical information for a given structure by computing both $S_{2}^{\left(w\right)}$ and $S_{2}^{\left(b\right)}$. Two-point probability functions possess certain properties, the most important of which are their physical realizability, i.e., the conditions at which a given function can be represented by a binary structure. Additional properties have been described in several comprehensive reviews [2,69].

Many other types of correlation functions exist: linear, cluster, chord distribution, and pore-size distribution, to name a few [2]. Each of them represents the probability that the position of either points or segments on the image satisfies some necessary conditions. Here, in addition to two-point probability functions, we utilize the linear function *L*_*2*_ (the probability that an entire line segment between two points belongs to one phase) and the two-point cluster function *C*_*2*_ (the probability that both ends of the line segments belong to the same cluster). The schematic representations of all three correlation functions used in this study are depicted in Fig 1. Both linear and cluster functions possess some non-trivial information about connectivity of the phases and, unlike the two-point probability function, do discriminate between phases. This means that $L_{2}^{\left(w\right)}$, $L_{2}^{\left(b\right)}$, as well as $C_{2}^{\left(w\right)}$ and $C_{2}^{\left(b\right)}$ are independent. The notion of averaging in case of statistically homogeneous (Eq 5) and isotropic structures (Eq 6) is also applicable for the linear and cluster functions.

There are several ways to calculate correlation functions for a given binary image. Ideally, for any two-point statistic, one establishes all possible connections between all points and then calculates average function values using, for example, Eq 6. This requires considerable memory and computing power, making simulated annealing optimization procedures (see below) very inefficient because resources increase enormously with increasing image size and dimension. A faster way to collect such statistics is based on Fast Fourier Transforms—FFT [70]. However, the FFT method works only for a two-point probability function. The original Yeong-Torquato method was implemented for two-point probability and linear functions [55], which were calculated along orthogonal directions by moving line segments across the image. As was demonstrated by Manwart and Hilfer [71], this approach resulted in artificial anisotropy in the diagonal directions. Jiao et al. [69] introduced the lattice-point method that can potentially handle any superposition of correlation functions, but as with all previous methods, it also averages function values over the whole image. Such approaches in calculating correlation functions cannot handle well anisotropic structures. Gerke et al. [68] have proposed a new method to account for structure anisotropy by calculating directional correlation functions. In order to calculate the above cluster function, *C*_2_, the binary phase of interest is first marked into clusters using the Hoshen-Kopelman algorithm [72] and a set of boundary conditions [73].

In the current paper two-point probability and linear functions are calculated in two orthogonal and two diagonal directions (as shown on Fig 1); cluster functions are calculated in two orthogonal directions only. Calculations involve moving line segments of varying length over the whole image, where the line segment ***r***(***x***_**1**_,***x***_**2**_) is as in Eq 5 for *S*_*2*_; the generalization for linear function *L*_*2*_ and cluster function *C*_*2*_ is straightforward. As a result, a relationship is obtained between average function value and length *r* in the direction of interest.

In this paper we apply correlation functions only to binary soil structures consisting of pores and solids. Correlation functions can be easily calculated and reconstructed for additional phases [74]. For example, in soil science multiple soil phases could include pores, mineral grains, clays and organic matter, each with their own correlation and cross-correlation functions [74–76].

### Reconstruction procedure

Provided with a set of correlation function(s), we will reconstruct soil structure by solving the inverse problem. Our method is based on the Yeong-Torquato technique which uses the so-called “simulated annealing” stochastic optimization algorithm [54] by matching correlation functions of a given realization with a reference structure by pixel permutations. This involves a set of two-point correlation functions in the form of $f_{2}^{\alpha }(r)$, where *α* is function type and ***r*** is defined similar to Eq 5. The difference between two realizations of the structure can be expressed as the sum of squared differences between two sets of correlation functions:

$$E=\underset{r}{\sum }\underset{\alpha }{\sum }{\left[f_{2}^{\alpha }\left(r\right)-{\hat{f}}_{2}^{\alpha }\left(r\right)\right]}^{2},$$ (10)

where $f_{2}^{\alpha }(r)$ and ${\hat{f}}_{2}^{\alpha }(r)$ are the values of the correlation functions for two different realizations (where the former is usually a value for an original, or reference structure, while the latter is for the structure being reconstructed). In Eq 10, *E* represents the "energy" of the system, which is minimized by the simulated annealing algorithm.

All modern soil structure measurement methods, including XMT or digital microscopy, result in digital pixel or voxel representation of the soil structure and are directly applicable for correlation function evaluation and reconstruction procedure implementation. At first, we create a random structure and start to change pixel positions (see further), while checking the system’s energy according to Eq 10. Because in the beginning of this process the characteristic sizes of phase aggregates are smaller than for the original image, it is reasonable to accept more permutations (i.e. changes in pixels’ position), even if they do not reduce the energy *E*. To this end, a so-called cooling schedule is chosen for the simulated annealing algorithm, which describes the probability of accepting any permutation *p* in the following way:

$$p\left(E_{old}\to E_{new}\right)=\{\begin{array}{c} 1,\Delta E<0\\ \exp\left(-\Delta E/T\right),\Delta E\geq 0 \end{array}\;,$$ (11)

where *T* is the "temperature" of the system, as interpreted from the Boltzmann distribution used in Eq 11 for Δ*E* ≥ 0, and

$$\Delta E=E_{new}-E_{old}.$$ (12)

The initial temperature *T* is chosen so that the probability *p* for Δ*E* ≥ 0 equals 0.5 [55,69]. The idea behind the cooling schedule is that simulated annealing will result in a global minimum energy *E*, and the optimization would not get trapped in some local minima. It is generally believed that a global minimum of *E* can be achieved when cooling is inversely proportional to the logarithm of *k*, i.e. *T*(*k*)∼1/ln(*k*), where *k* is a number of permutation trials. Several complex cooling schedules exist which depend on numerous system parameters [63]. In practice, a faster cooling schedule is usually utilized in a form of *T*(*k*)/*T*(0)∼*λ* ^*k*^, where *λ* is the annealing scheduling parameter with a value somewhat smaller than, but close to unity [55,69]. Based on numerous trials and experience in reconstructing different test cases [60,67–68], we choose to implement a slower cooling schedule based on the following geometrical progression:

$$T\left(k\right)=T\left(0\right)\lambda^{\left(k-1\right)}.$$ (13)

The simulated annealing algorithm is a time-consuming and computationally expensive technique, although several ways exist for its optimization and acceleration. For example, the algorithm can be potentially parallelized using e.g. the mixing-of-states method [77], but to date we are not aware of published reconstruction techniques utilizing some means of parallelization. Another acceleration technique involves optimization of correlation function evaluation and is based on the idea that only subsets of correlation functions need recalculation for each permutation step [55,69,74]. Yet another way to speed up the procedure is by improving pixel permutation. Random selection of a pair of pixels for permutation will result in very ineffective choices, especially at the late stages of the reconstruction (i.e. approaching minimum level of energy) where it is important to select isolated elements and preferably join them with the corresponding phase [78].

Finally, computational efforts can be further reduced if the optimization is based on limiting the length of the segments used for calculating correlation functions, e.g., by using a cut-off distance |***r***| as in Eq 5. Ideally, such length should be larger than the average size of structure elements in the image [69], and can be evaluated from the original set of correlation functions (see examples in S1 File) ensuring correlation functions maintain a significant part of their asymptotic behaviour. The maximum value assigned to the cut-off distance |***r***| should be equal or smaller than the size of the original image.

Here we utilize a relatively simple optimization method in choosing permutations, thereby closely following Čapek et al. [63]: 1) choose a random location within a phase of interest, 2) choose two random directions, and 3) in each of these directions, choose two pair of pixels with a minimum distance between them such that they satisfy two additional conditions: a) pixels lie in opposite phases, and b) pixels lie at the interface. A purpose-built optimization algorithm for recalculating S_2_ and L_2_ functions during annealing is used, which involved application of periodic boundary conditions (i.e. opposite sides of image boundaries are connected) for correlation functions’ evaluation during the reconstruction procedure.

The original Yeong-Torquato technique for image reconstruction based on correlation functions calculates *S*_*2*_ and *L*_*2*_ only along orthogonal directions. In later work, diagonal directions were added [63,71], but as with all correlation-based methods used to date, average statistics are determined based on all directions used for correlation function evaluation. Significant improvement in quality of reconstruction can be achieved by calculating correlations in directions, but without averaging across directions to describe anisotropic structures [68,79]. During reconstruction, each direction for each function is included separately in Eq 10. We use *S*_*2*_ and *L*_*2*_ functions for reconstructions, and calculate *C*_*2*_ to verify the quality of the resulting images by comparing its energy *E* for originals and reconstructions. The cluster function was previously shown to be a superior descriptor [80] and resulted in very accurate reconstructions for isotropic simple binary structures. However, the optimization technique involving the *C*_2_ correlation function is not as efficient as the *S*_*2*_ and *L*_*2*_ functions because of the requirements to track cluster dynamics after each permutation [81]. This requirement makes routine stochastic reconstructions of large 2/3D images using *C*_*2*_ computationally very expensive.

**Fig 2:**
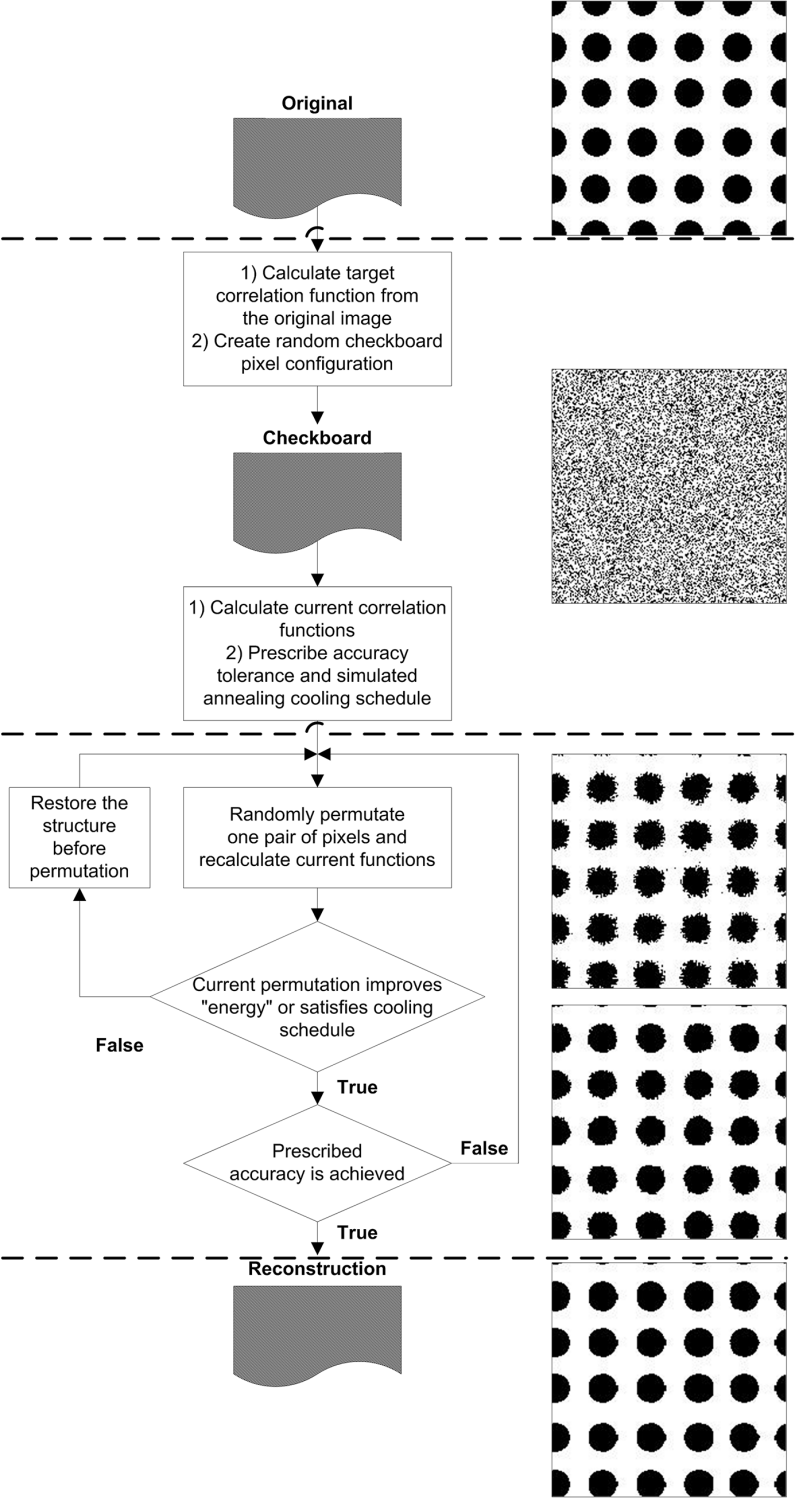
Overall scheme of the reconstruction procedure. Illustrations are provided for each stage using reconstruction of circles as example.

In summary, reconstructing images first requires correlation function(s) to be calculated from the original image and stored as a “reference” (Fig 2). In the next step, we create a random image—a so-called checkerboard—with phase fractions (e.g. porosity) that are known directly from the reference correlation function according to Eq 7, and subsequently calculate and store its correlation function(s). The process of random permutation then proceeds by choosing two pixels of different phases and calculating a change in correlation function after their permutation; we either accept or reject the permutation according to Eq 11. After that, another pair of pixels is evaluated for permutation, seeking to reduce the energy of the image. This loop of permutations and correlation function recalculations should be stopped at some point to finalize the reconstruction procedure. There are two popular ways to do that: 1) after some number of consecutive unaccepted permutations according to Eq 11 (for example, 10^6^), or 2) by choosing some accuracy threshold value for the energy *E*. The latter method is preferable, as it allows the comparison of different reconstructions as for similar size and porosity images similar *E* values will mean similar accuracy [82]. The scheme of the reconstruction procedure with simple examples is depicted on Fig 2.

### Morphological analysis

**Fig 3:**
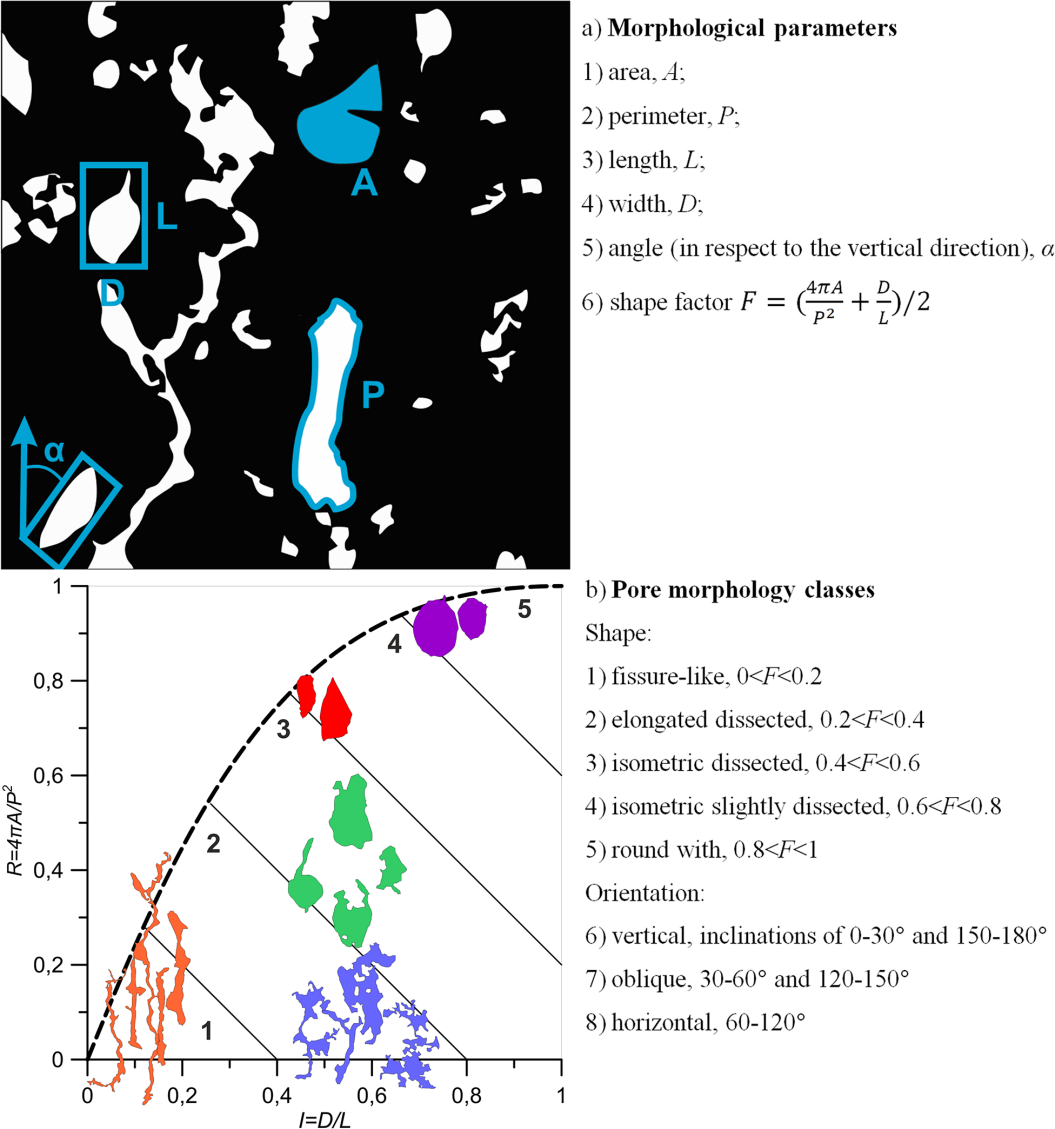
Main concepts of the morphological analysis. a) morphological parameters calculated for each pore element, and b) examples of pores extracted from original soil images and their shape classifications (all five shape classes are shown in roundness (*4πA/P* ^*2*^)—isometry (*D/L*) coordinates).

To compare thin-sections and their correlation function-based reconstruction, we apply morphological analysis to both original images and their replicas. We first define a pore on a 2D image as a cluster of connected non-solid material (i.e., white areas on all binary images). All pores obtained in such a manner are marked and stored separately. For each pore, five different parameters are calculated (see Fig 3A for a schematic representation of all parameters): 1) area *A*, 2) perimeter *P*, 3) length *L*, defined as the longest side of the circumscribing rectangular, 4) width *D*, the shortest side of the circumscribing rectangular, and 5) orientation index, expressed as angle *α* between the longest side of the circumscribing rectangular and the Y-axis. Based on the first four parameters, the following shape factor *F* is defined [41]:

$$F=\left(\frac{4\pi A}{P^{2}}+\frac{D}{L}\right)/2,$$ (14)

where the first element in Eq 14 refers to the so-called object roundness, and the second element characterizes the pore isometry. The shape factor *F* as defined by Eq 14 takes on values between zero and one and has several advantages over more commonly used definitions of roundness, such as the squared perimeter *P* ^2^ [40]. One such advantage is that it allows distinguishing between round pores and fissure-like pores, as well as a broad range of other possible shapes (Fig 3B).

In addition to calculation of the above morphological measures, the total number of the pores is reported for each image analyzed. Based on the main five morphological parameters and elements of the shape factor *F*, each pore is classified into one of five classes according to its shape (defined by parameter roundness and isometry) and one of three classes of orientation (defined by parameter *α*). The five shape classes are as follows (Fig 3B): 1) fissure-like with 0 < *F* < 0.2, 2) elongated dissected with 0.2 < *F* <0.4, 3) isometric dissected with 0.4 < *F* < 0.6, 4) isometric slightly dissected 0.6 < *F* < 0.8, and 5) round with 0.8 < *F* < 1, 6). The three orientation classes are (Fig 3B): 1) vertical, with inclinations of 0–30° and 150–180°, 2) oblique, with inclinations between 30–60° and 120–150°, and 3) horizontal, with inclinations between 60–120° All eight classes together are used for comparison of original and reconstructed images (see further).

### Soil thin-section images, analysis and reconstruction procedure

In order to study reconstruction techniques and test applicability of correlation functions to describe soil structure, we have chosen eight 2.1x2.1 cm^2^ soil thin-section binary images (segmented into solid material and pores) of soils of the Russian Plane (Table 1). These soil types (images) are marked I-VIII. All images had the size of 994 by 994 pixels with a resolution of 21.2 μm.

**Table 1:** Soil thin-section information.

Structure type	Soil type*	Horizon	Sampling depth, cm
**I**	Soddy-podzolic	C (parent material)	170–180
**II**	Chernozem	C (parent material)	170–180
**III**	Grey forest	BC (transitional horizon)	80–90
**IV**	Chernozem	Ap (ploughed humus horizon)	5–10
**V**	Chernozem	A (humus horizon)	5–10
**VI**	Grey forest	B (illuvial horizon)	60–70
**VII**	Soddy-podzolic	EL (eluvial horizon)	20–25
**VIII**	Podzol	EL (eluvial horizon)	20–25

*according to Russian soil classification [83]

**Fig 4:**
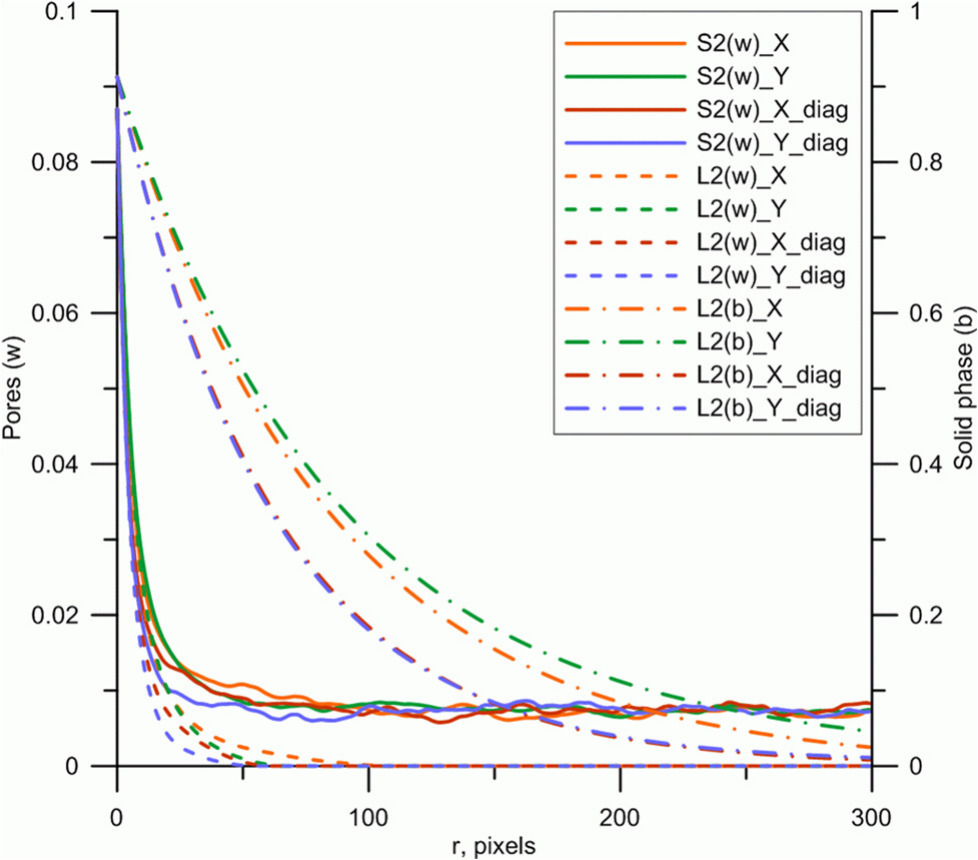
Correlation functions for pores (solid and dashed lines) and solid phase (dash dot line). ***S***_***2***_^***(w)***^ **and *L***_***2***_^***(w)***^ **are, respectively, two-point probability and linear functions for pore phase; *L***_***2***_^***(b)***^ **is a linear function for solid phase.** All correlation functions are evaluated in four principal directions according to Fig 1. Example is for soil type I exhibiting the largest spatial correlation length of *L*_***2***_^*(b)*^ across all soil types.

In total five reconstructions were made for each thin-section image. This involved using two-point probability functions for pores and two-point linear path functions for both pores and solid phases; these correlation functions provide a new way for quantifying soil structure. All functions were computed in the main orthogonal and diagonal directions (i.e. four directions in total), based on a recently developed procedure [68]. The size of reconstructions was the same as of original images, i.e. 994^2^ pixels. The reconstruction procedure was based on the following parameters: 1) the annealing schedule parameter *λ* = 0.999999, a value which ensures slow annealing cooling while reaching ground state (i.e. with global minimum energy or best fit between original and reconstructed image) according to Eq 10; 2) the spatial cut-off is at |***r***| = 300 pixels to capture all correlation lengths on the original soil images (based on example soil type I displaying the overall slowest decay of the *L*_*2*_^*(b)*^ function, Fig 4); 3) a prescribed accuracy *E* = 10^−7^ was used to terminate the iterative reconstruction procedure, which roughly corresponds to leaving about 1–2% of all pixels that are ill-positioned and therefore contribute to a mismatch between original and reconstructed correlation functions [67,82]. Strictly speaking, because of the stochastic permutations invoked for the annealing procedure, no parameter fitting or optimization of reconstructed structures is made at any stage. Also note that the reconstruction procedure preserves total porosity, i.e. originals and reconstructions are always identical in terms of porosity, as the first moment of any correlation function for a given phase is equal to the phase’s fraction (Eq 7) [2].

All images, including each original thin-section and its five reconstructions, were analyzed in terms of a solid space and a pore space—structure, using the different metrics for further qualitative comparison. First, for each soil type comparison was performed on the basis of the total number of pores and the pore-size distribution of an entire image. Next, directional cluster functions were calculated in two orthogonal directions for all images. The comparison was performed by computing energy similar to Eq 10 (squared difference between cluster functions of original and reconstruction):

$$E=\underset{r}{\sum }\underset{\alpha }{\sum }{\left[C_{2}^{\alpha }\left(r\right)-{\hat{C}}_{2}^{\alpha }\left(r\right)\right]}^{2},$$ (15)

where $C_{2}^{\alpha }(r)$ and ${\hat{C}}_{2}^{\alpha }(r)$ are the values of the correlation functions for reconstruction and original image, respectively, and summation with *α* goes over two orthogonal directions (horizontal and vertical) and two phases (black and white). Finally, morphological analysis of each image is performed as described above and the number of pores falling into each of eight morphology classes (based on Fig 3B) is calculated. Based on the sum of squared differences (SSD) calculated for each parameter analyzed, the best reconstruction (i.e. lowest SSD) for each soil type was identified. On the basis of these criteria, which each highlight a different feature of soil structure, an evaluation of reconstructions can be made.

## Results and Discussion

**Fig 5:**
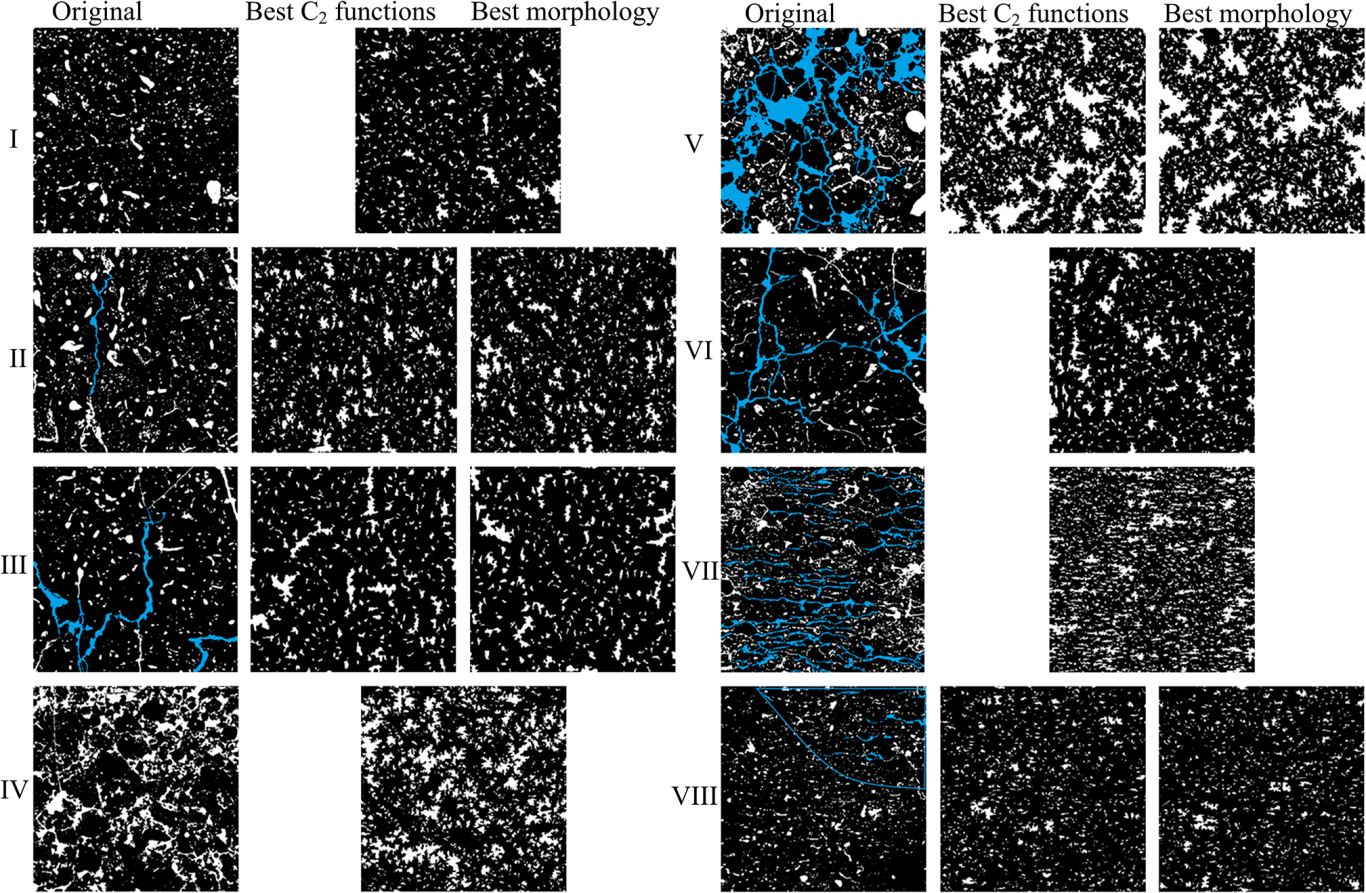
All original eight soil type images (left column) with their best performing reconstructions based on a cluster function analysis (middle column) or pore morphological analysis (right column) (if reconstruction performance for both analyses is identical, then only one image is shown). Size of thin section = 2.1×2.1 cm^*2*^. Blue shaded areas highlight pore features that were poorly reconstructed: type II) vertical pore; III) complex elongated pores; V) one connected pore dominating entire image; VI) one connected fissure-like pore; VII) numerous horizontal cracks; VIII) horizontal features in the upper-right marked region.

**Fig 6:**
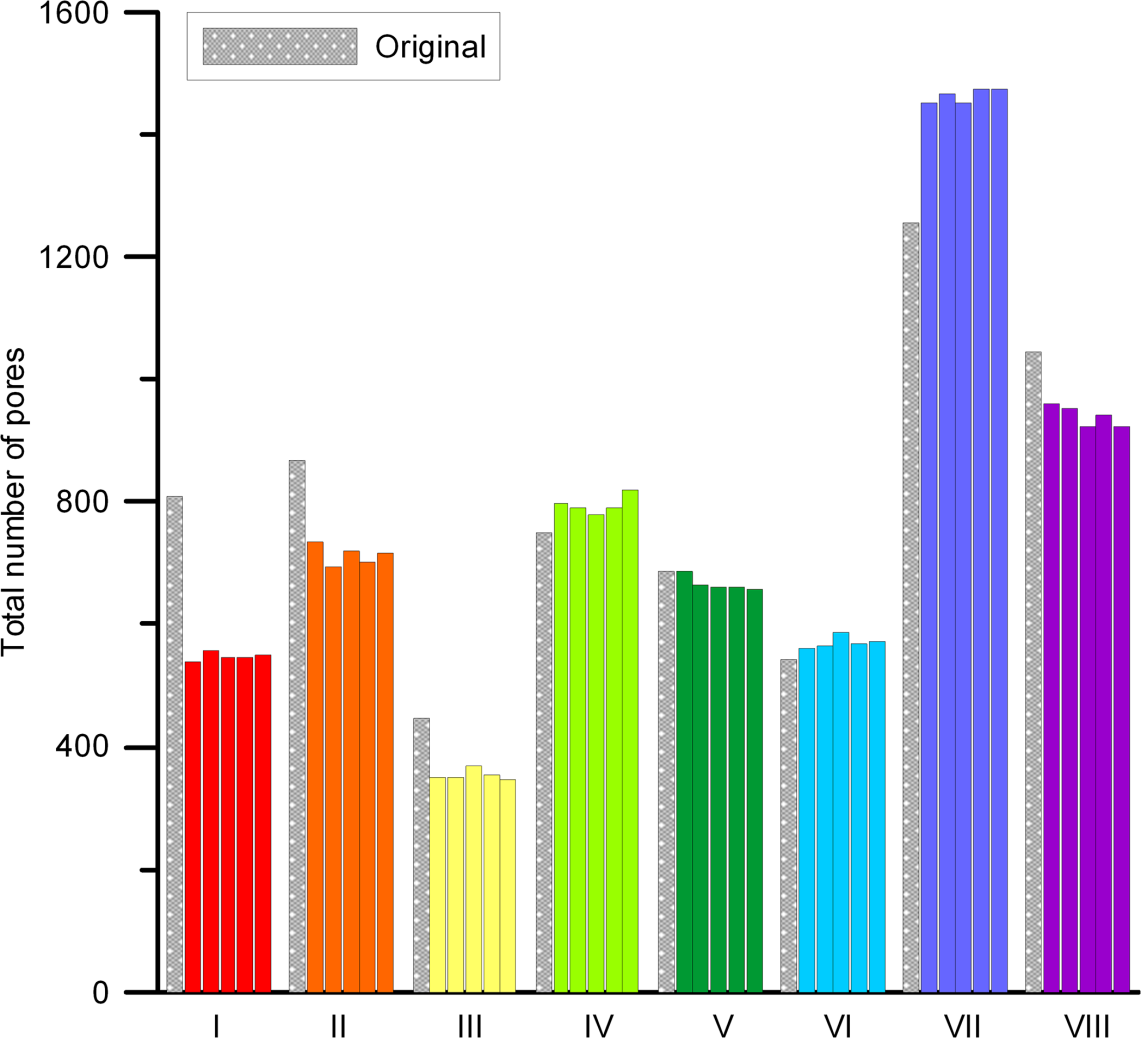
Total number of pores for original images and their five reconstructions compared for all soil types (I-VIII).

**Fig 7:**
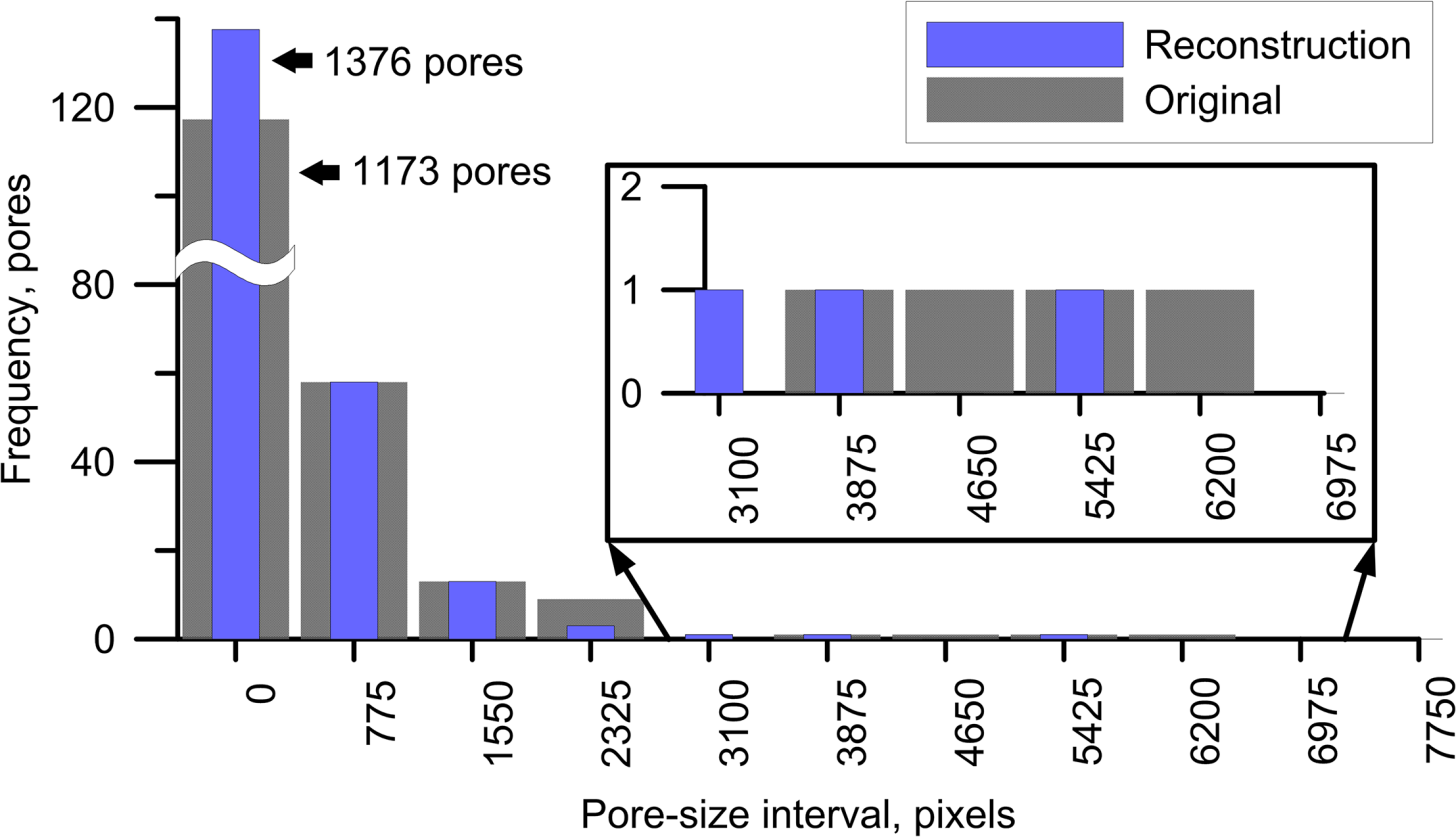
Comparison between pore-size distribution for original soil type VII and its corresponding best reconstructed image according to pore morphological metrics (a larger pore range is zoomed out for better visibility of the resulting distribution).

A visual comparison of all eight original images and their best performing reconstructions suggests that reconstructions are broadly capable to reproduce the main features of the original soil images, including anisotropic structures and patterns of pore aggregates (Fig 5, to see all five reconstructions for each soil type, refer to S1 File for this article). Obvious flaws in the reconstruction are 1) inability to correctly reproduce elongate features such as cracks and crack-like connecting pores for soil types II, III, V and VI, and 2) somewhat shorter reconstruction of the horizontally oriented features for soil type VII. The total number of pores (Fig 6) and the pore-size distributions (Fig 7 and S1 File) generally show a relatively good agreement between original thin-sections and the reconstructions. All reconstructions resulted in a nearly perfect match between the original and target correlation functions.

**Fig 8:**
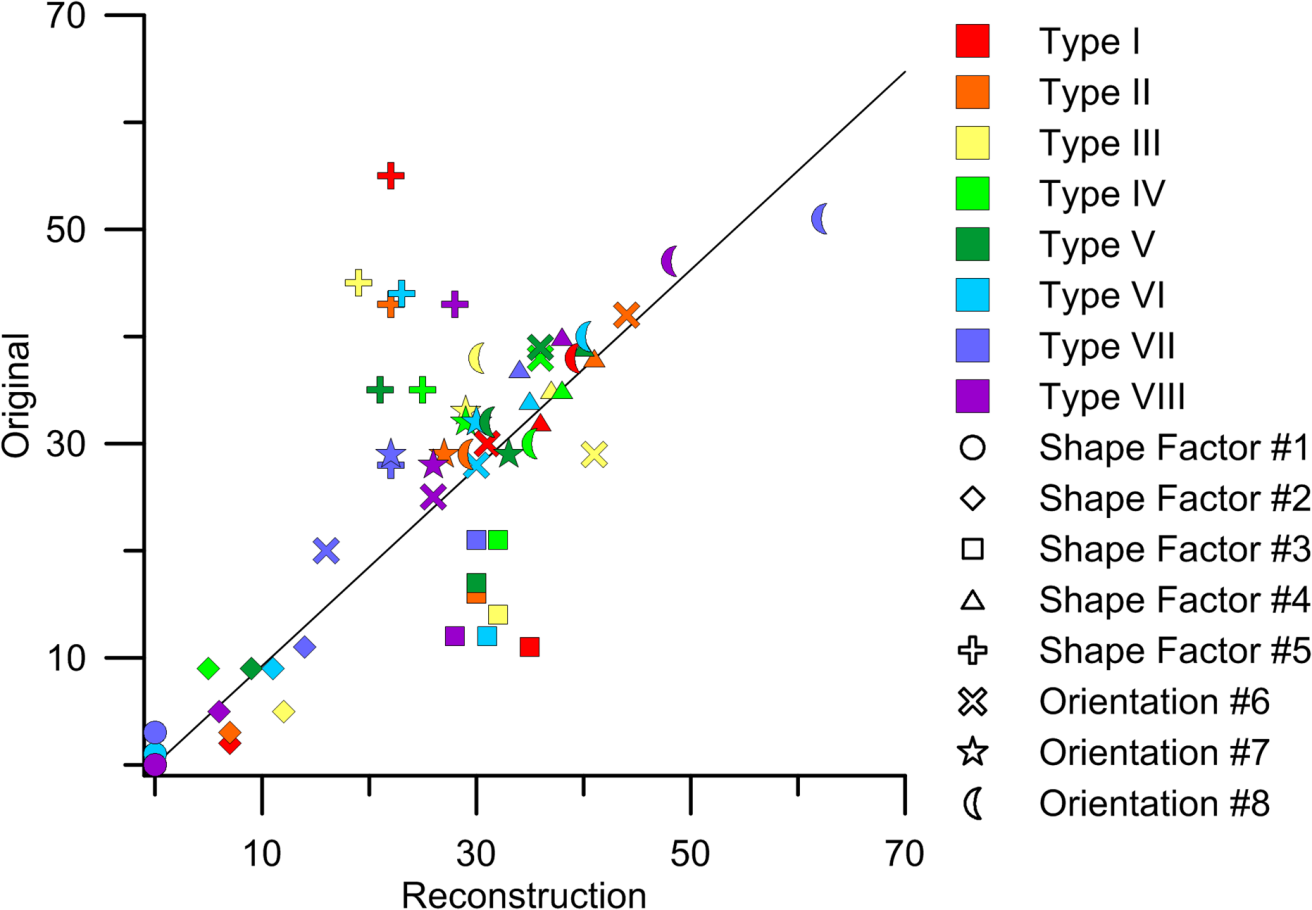
Scatter plot of pore morphology classes using pore shape class 1–5 (Fig 3B) and orientation classes 6–8 (Fig 3B) for original and best reconstructed images for all soil types I-VIII.

The morphological analysis further showed a good agreement between original and reconstructed pore shape parameters and their orientation metrics (Fig 8). Most prominent deviations can be seen for circular and isometric dissected pores (shape factor 5 and 3). For the former, the numbers are usually much lower on reconstructions. This is mainly due to the presence of mixed shaped pores on all original images, for which the correlation functions, when averaged over different directions, cannot capture roundness appropriately (unlike in the case when all structures are round, like on Fig 2). For this reason, the number of class 3 pores (isometric dissected) was much higher on replicas. This effect for classes 3 and 5 is especially pronounced for soil type I, where the majority of pores in the original image are round. All pore orientations (classes 6–8) were captured accurately by functions computed only in four (two orthogonal and two diagonal) directions. This means that the method is sufficiently applicable for anisotropic structures. In other words, directional correlation functions can identify and describe anisotropic soil structures.

**Fig 9:**
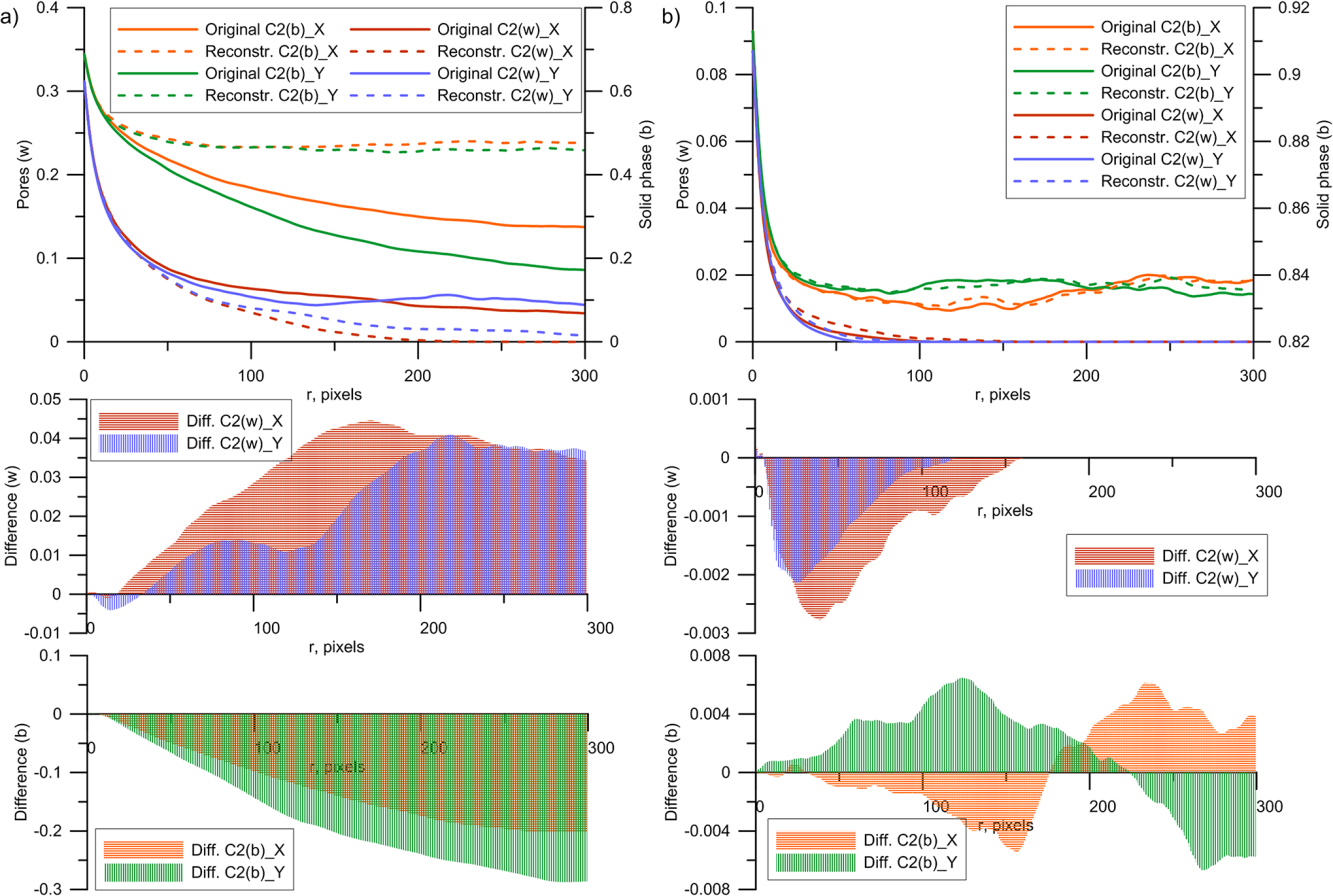
Comparison of *C*_*2*_ cluster functions for original and reconstructed soil images for a) soil type I (best case), and b) soil type V (worst case). The legend is similar to that of Fig 4 for two-point probability and linear functions.

**Fig 10:**
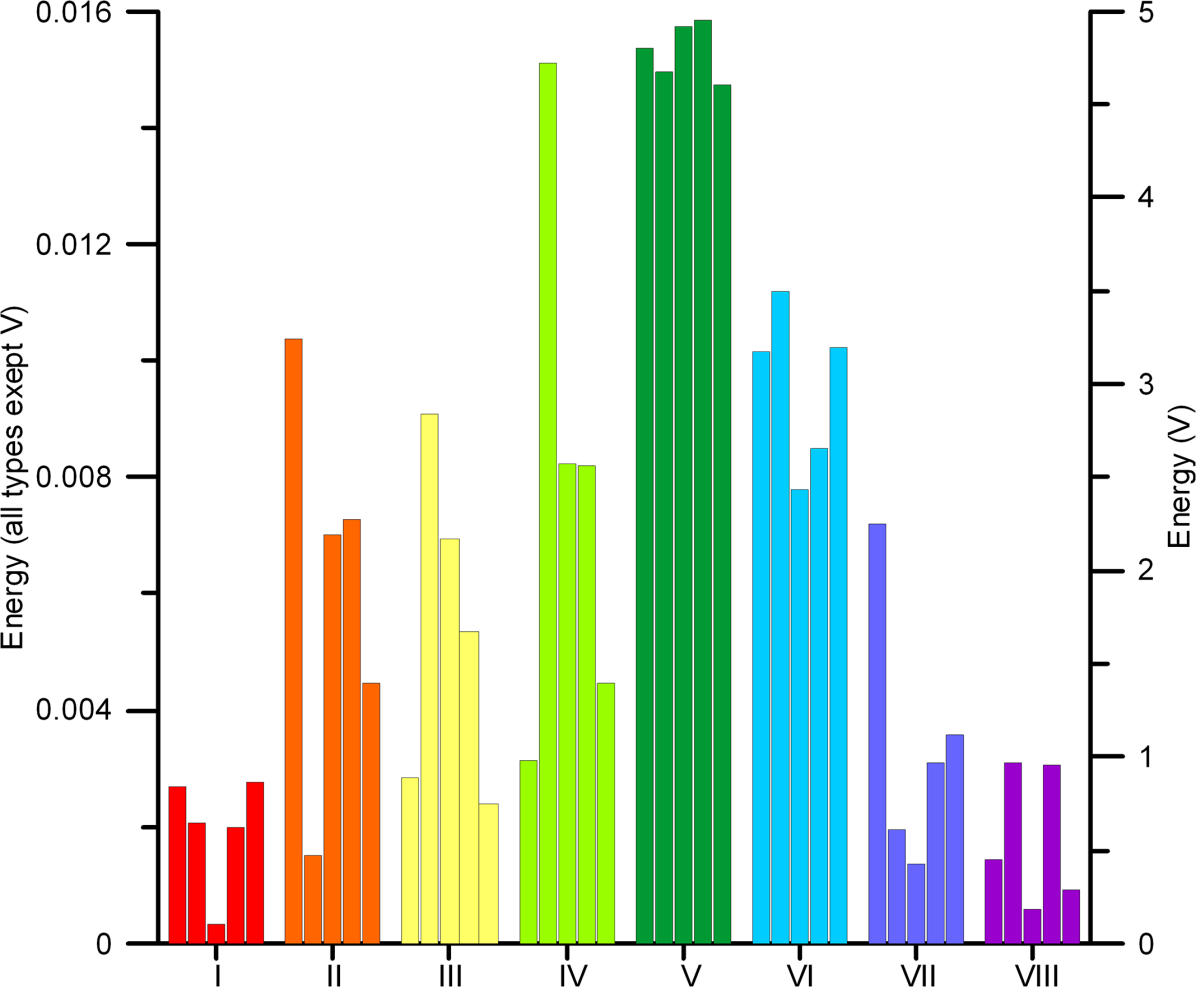
Cluster function differences for all reconstructed soil images (calculated as arithmetic average of *C*_*2*_ differences between originals and replicas for each soil type and each orthogonal direction).

Three sets of metrics were applied to test the quality of the reconstruction algorithm. First, the best out of five reconstructions for each of eight soil types was chosen based on a series of morphological parameters (Fig 3) and evaluation of squared differences (marked with * on histograms for all soil types in S1 File). Next, squared differences similar to energy *E* were calculated with cluster functions obtained from all original images and replicas (Figs 9 and 10); this allows selecting the best reconstruction according to connectivity statistics. Finally, the best reconstruction was also determined by comparison of the total number of pores (Fig 6) and pore-size distributions (Fig 7). Table 2 summarizes the results of comparing these three different sets of metrics; in most cases the total number of pores and the pore-size criteria provided similar results, while for six soil types (marked with *) the best reconstruction according to either morphology and cluster, or cluster and pore statistics, coincided. This indicates some degree of consistency between the metrics applied, hence adds confidence to the selected “best” reconstruction. On the other hand, observed disagreements also highlight the limitations of the applied metrics as unique identifiers of pore structure and as a comparison tool to assess reconstruction quality. As the prime interest of studying soil structure is to obtain biophysical soil properties, the best criterion would be one based on a comparison of such physical properties for original and reconstructed images, which typically requires three-dimensional reconstructions and pore-scale modeling [84–85].

**Table 2:** Summary of best reconstructions according to different quantitative metrics (numbers 1, 2, 3, 4, or 5 refer to reconstruction method, available in S1 File).

Metrics	Soil type							
	I	II	III	IV	V	VI	VII	VIII
**Pore size distribution**	2	1*	3*	4	1	1	1	1
**Total # of pores**	2	1*	3*	3	1	1	1,3*	1
**Cluster function C**_**2**_^**(b)**^ **, C**_**2**_^**(w)**^	3*	2	5	1*	5	3*	3*	3
**Morphology (eight classes)**	3*	1*	3*	1*	3	3*	3*	5

Cluster functions calculated separately for original and reconstructed images showed acceptable reconstruction quality (i.e. displaying very similar key structural features) for most soil types (see Fig 9 and S1 File). A nearly perfect match between original and reconstructed cluster functions was obtained for soil type I; this soil is characterized by a single disconnected porosity. Similar observations are made for soil type IV, which also consists of isometric, disconnected agglomerates of pores (S1 File). A further good agreement is observed for types II and VIII (S1 File). Their cluster function values deviate only slightly for reconstructed pore space (white): i.e., the reconstruction is lower in the vertical direction due to the inability to reconstruct a vertical crack on the original image (Fig 5) for soil II. The observed higher connectivity for soil type VIII is due to presence of the area with horizontal cracks. Some significant deviations can be seen for the pore phase (but still good agreement for solid phase) of soil types III and VII: with lower connectivity for reconstructions in horizontal direction due to the absence of mainly vertical cracks present on the original image for soil III, and presence of horizontal elongated pores for soil VII (highly connected horizontal cracks, Fig 5). The worst performance according to cluster functions metrics is observed for soil type V, where the original pore space is highly connected via small cracks (creating one large interconnected pore highlighted on Fig 5), a feature which is much less present on the reconstructions. A lower degree of connected pore space is also visible for soil type VI. All *S*_*2*_, *L*_*2*_ and *C*_*2*_ functions provide complementary information on structure anisotropy; however, the degree of anisotropy measured by each function can be different and suggests that different correlation functions do provide additional and important information on soil structure.

Each reconstruction of the 944 by 944 pixel images required approximately 0.3 to 1.5 hours of program execution time on a Intel Xeon X7560 2.26 GHz CPU. This includes up to 10 minutes for the calculation of the *S*_*2*_ *-L*_*2*_ *-C*_*2*_ correlation functions set. This means that such correlation functions can be easily evaluated for standard soil images, while the reconstruction procedure for 2D images is sufficiently fast to be performed on a personal computer.

The inability of the *S*_*2*_ *-L*_*2*_ correlation functions to reconstruct connectivity for soil types V and VI highlights some of their current limitations for soil structure characterization and reveal a necessity to include more correlation functions into soil structure description and reconstruction. We would like to point out that the reconstruction procedure was implemented here mainly to demonstrate that the structural features can be reconstructed using correlation functions and also highlight the importance of information content (discussed below). In this sense, correlation functions are more powerful than any other proxy soil structure descriptor known to date (e.g., grain-size distribution, pore-size distribution, Minkowski functionals, water retention curve, etc.). Significant improvement is observed here in comparison with our preliminary application of correlation functions (averaged two-point probability function only) to structure description by using the original Yeong-Torquato technique [67]. To the best of our knowledge, this is the first application of directional correlation functions to reconstruct anisotropic soil structure.

Several types of discrepancies were evident from the above analysis. The discrepancies are especially important as concerns the connectivity of the pore space, which is consistently lower for the reconstructed image. This in turn results in a different number of total pores and different pore-size distributions, with the degree of discrepancy depending on the soil type (see S1 File). This can be explained in part by 1) the fact that the set of correlation functions employed contains insufficient information about pore structure [86], and in part by 2) the inappropriate weighing of different correlation functions employed based on their information content according to Eq 10 [68]. The remaining challenge, thus, is to determine which sets of correlation functions contain enough information to characterize a given soil structure. The existence of degenerated states of the *S*_*2*_ correlation function was demonstrated for hypothetical structures and some simple composite materials [86–87]. Furthermore, reconstructions using only the *S*_*2*_ correlation functions have been shown to result in a less connected pore space [88–89]. The behavior of the *L*_*2*_ and *C*_*2*_ correlation functions in regards to their degeneration properties is an active area of research in theoretical physics. However, while *S*_*2*_ and *L*_*2*_ based isotropic reconstructions do work well for some simple heterogeneous materials such as composites and sintered filters [2,63,90], it is clear from our study that their applicability to reconstruct complicated soil structure is limited.

Adding more sampling directions for correlation functions involved in the reconstruction scheme could help to improve reconstruction quality. However, applying a large number of directions would increase the computational resources significantly. The result is slow convergence of the resulting information content to a 100% one is expected with increasing number of directions, similar to the use of higher-order correlation functions [86]. Another potential way to improve reconstruction of images exhibiting highly connected pore spaces would be the use of the erosion-dilation method [81,91]. A third way to improve reconstruction quality is implementation of the *C*_*2*_ correlation function for reconstruction procedures, as it should connect all disconnected pore aggregates for types V and VI, as well as improve statistics for all other types. We note again that the *C*_*2*_ correlation function was calculated here only to assess the degree to which the reconstructed image matched the original one. Also, note that cluster functions for the same soil sample measured in 2D and 3D are fundamentally different; only the latter represents true connectivity information. The use of the *C*_*2*_ correlation function for reconstruction purposes is currently too computationally intensive; to date it has been applied only for some simple test cases and Al-Si-Fe alloy images of limited size [79–80].

Another likely reason why our reconstructions failed to capture particular features, such as elongated pores, is statistical inhomogeneity or non-stationarity of soil structure. According to the definition in Torquato [2], "the media is statistically homogeneous if the joint probability distributions describing the stochastic process are translationally invariant, i.e., invariant under a translation (shift) of the space origin". In other words, such distributions are independent of position. Some areas with anisotropic, i.e. elongated pores are significantly different from the rest of the pore space (highlighted in blue on Fig 5); other typical examples of statistical inhomogeneity observed in our soil thin-section images include two types of porosities within the same image, e.g. regular round and fissure-like pores (see Fig 5). This means that transition from Eq 5 to Eq 6, i.e. the assumption about statistical homogeneity, is not exactly justified. Correlation functions have been used by numerous researchers to reconstruct many different heterogeneous materials; however, the statistical homogeneity of the input images was rarely checked [55,58,60,62–63,67,79]. To the best of our knowledge, to what degree the combined *S*_*2*_ *-L*_*2*_ *-C*_*2*_ correlation functions can address the problem of statistical inhomogeneity or non-stationarity has not been tested; only *C*_*2*_ in combination with *S*_*2*_ was applied in reconstructions of very simple cases [79–80]. Periodic structures can be reconstructed exactly [68,75], but they are rarely, if ever, observed in soils and other natural porous media.

Correlation functions combined with simulated annealing are not the only reconstruction procedure that can be used to create 2/3D images from 2D cuts. Other methods include: 1) Gaussian random fields [92], multi-point statistics (MPS) [85,93–96], entropic descriptors [97], fractal dimension measures [49], and process-based algorithms [50–52]. MPS is one of the most popular methods and was recently proven to be very effective in reconstructing heterogeneous porous media [85,95–96]. Applications of MPS to soil include 2D [93] and 3D [84,98] Markov chain reconstructions, a variation of the multi-point method. However, such methods do not provide any information on porous media structure per se, as they usually operate with probabilities of image events in a window of given size, or reconstruct images using the mosaic from the original.

In addition to being used for structure quantification and stochastic reconstructions, correlation functions can also be applied to evaluate numerous physical properties using so-called rigorous bounds [2]. Current methods mainly include the usage of three-point or four-point probability functions S_3_ and S_4_ [99]. However, the performance of such methods was rarely demonstrated for soils. As it was shown that S_3_ and S_4_ provide insufficient information to fully characterize porous media in terms of structural properties [80,86], we expect insufficient accuracy from applying rigorous bounds involving S_3_ and S_4_. Nonetheless, such methods can find their use in providing approximate estimations of soil properties in a computationally efficient way.

The importance to soil science of porous media structure description and reconstruction algorithms based on correlation functions should not be underestimated. Examples include: 1) reconstructing 3D data from digitized thin-sections; 2) describing spatial correlations for minerals, clays, organic matter, microbial activity in soil and their temporal dynamics following treatment or management options; 3) monitoring soil degradation processes; and 4) soil classification. Potentially reconstructions can be also used to derive soil hydrophysical properties such as hydraulic conductivity, water retention properties and relative permeabilities for unsaturated air/water flow [5]. The latter involves reconstructing 3D soil images from 2D cuts [100–101] followed by pore-scale fluid flow modeling. Developing 3D reconstructions by applying 2D correlation functions is especially appealing for soils, as they often have different pore structures at different scales such that an integrated multi-scale 3D analysis may become prohibitive. For example, nano-scale porosity can be characterized using FIB-SEM/SEM 2D imaging from which 3D reconstructions can be developed [60]. Macro-porosity can then be characterized using lower resolution XMT; assemblage of the nano- and macro-porosity is the final step in achieving a consistent multi-scale pore system [60,102].

In this article we focused on soil structure in 2D, as both the thin-section measurements and the reconstruction of soil structure using correlation functions provided 2D information only. Establishing 3D reconstructions of soil structure from 2D thin-sections would be a logic next step, and would be particularly valuable in the case of anisotropy in more than one direction. It can also form the basis for permeability prediction using flow simulation [67,84]. Even when the spatial domain is limited to 2D, the testing of correlation function-based reconstruction algorithms is still relevant: it ensures an optimal method can be identified in a stepwise manner, i.e. in 2D first when complexity and computational resources are less than in 3D.

According to our current findings, the main questions we need to answer before correlation functions can be applied with confidence to address today’s grand challenges in soil science are as follows:

1. Are sets of *S*_*2*_ *-L*_*2*_ *-C*_*2*_ correlation functions unique for each soil type?
2. How will the introduction of the *C*_*2*_ function into reconstruction procedures affect reconstruction quality?
3. What set of correlation functions is necessary to reliably specify soil structure?
4. What soil properties can be reliably predicted using correlation functions and reconstructions?
5. Can we use correlation functions to describe soil structure dynamics as a result of freezing/thawing or wetting/drying cycles?
6. What degree of comprehensiveness of statistical information is provided by calculation of correlation functions in multiple directions, and what is a minimum of such directions?

Answering all ore even a few of these questions may significantly improve our ability to describe soil structure, and its dynamics.

## Conclusions

In this paper we explored the performance of universal correlation functions in characterizing and reconstructing soil binary (solid-pore phases) structures. In particular, for the first time, we applied two-point probability and linear correlation functions to both characterize and reconstruct 2D soil images. In the current analysis, cluster correlation functions were introduced to better characterize soil connectivity and compare quality of reconstruction schemes. Also for the first time, correlation functions were computed in four directions and applied to natural porous media (soil). Based on two-point probability functions and linear correlation functions calculated from original soil thin-section images, we reconstructed 2D soil structure using a simulated annealing optimization technique. Future improvements of this novel approach will help to overcome the current limitations in regard to reconstructing soil structure.

In reconstructing eight different soil types with contrasting pore structures, major differences between reconstructed and original image were noted and interpreted as a lack of information content in the correlation functions employed. We compared original thin-sections and their stochastic reconstructions using three sets of test metrics. Set one involves an original morphological analysis. Set two used cluster correlation function computations, while the third set uses the total number of pores and their pore size-distributions.

None of these metrics were found to be sufficient to uniquely characterize the difference between original and reconstructed images. This also demonstrates that the conventional measures such as pore-size distribution are insufficient to characterize soil structure. Possibly the best way to measure the accuracy of reconstruction would be to apply pore-scale modeling approaches to determine physical properties (such as *K*_*sat*_ or water retention curve) of 3D reconstructed soil structures and compare them to independent laboratory measurements.

The two-point probability, linear and cluster correlation functions showed potential to accurately describe structural properties for both solid and pore phases of all thin-sections for all eight soils; moreover, they can be parameterized with a limited number of fitting parameters or basis functions. This provides numerous opportunities for future applications of correlation functions in soil science, which may include soil classification, degradation monitoring, spatial description of microbial activity, to name only a few.

## Supporting Information

S1 File A full dataset of reconstruction and analysis results. In this material for each of eight soil types we report: 1) original image of the thin-section, 2) all five reconstructions obtained using the method described in paper, 3) a set of *S*_*2*_ *-L*_*2*_ correlation functions for original image (all reconstructions have similar correlation functions up to a tolerance of *E* = 10^–7^, all minor differences would be invisible on the such a graph), 4) a comparison of cluster functions computed for original image and best replica judging by differences in cluster function values, 5) comparison of pore-size distributions for original thin-section and the best replica judging by morphology analysis, 6) a table with results of morphological analysis covering all replicas and the original.
(PDF)
