# Supplementary material for: Universal Spatial Correlation Functions for Describing and Reconstructing Soil Microstructure
Source: PLoS One. 2015 May 26;10(5):e0126515. doi: 10.1371/journal.pone.0126515 (PMC4444105; doi:10.1371/journal.pone.0126515)

Supplementary materials for the manuscript " *Universal spatial correlation functions for describing and reconstructing soil microstructure* " by Marina V. Karsanina, Kirill M. Gerke, Elena B. Skvortsova, and Dirk Mallants

File S1. A full dataset of reconstruction and analysis results. In this material for each of eight soil types we report: 1) original image of the thin-section, 2) all five reconstructions obtained using the method described in paper, 3) a set of S_2-L_2 correlation functions for original image (all reconstructions have similar correlation functions up to a tolerance of E=10^{-7}, all minor differences would be invisible on the such a graph), 4) a comparison of cluster functions computed for original image and best replica judging by differences in cluster function values, 5) comparison of pore-size distributions for original thin-section and the best replica judging by morphology analysis, 6) a table with results of morphological analysis covering all replicas and the original.

For more details on correlation functions computation, reconstruction procedure and morphological analysis, please, refer to the original paper.

Type I

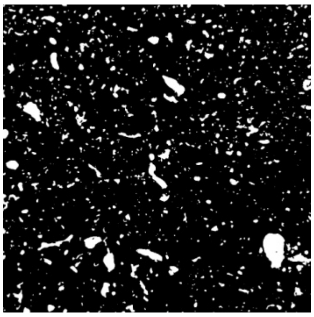

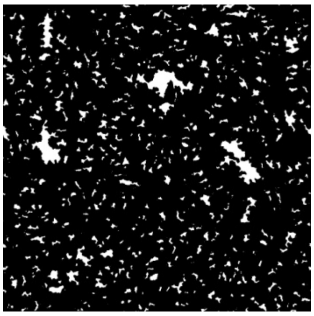

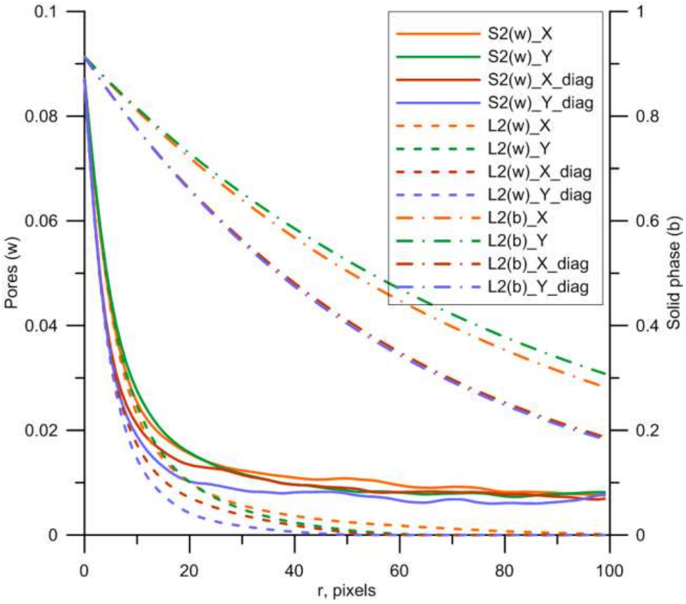

1

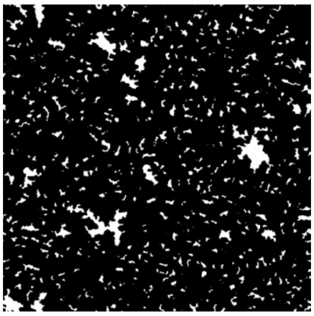

2

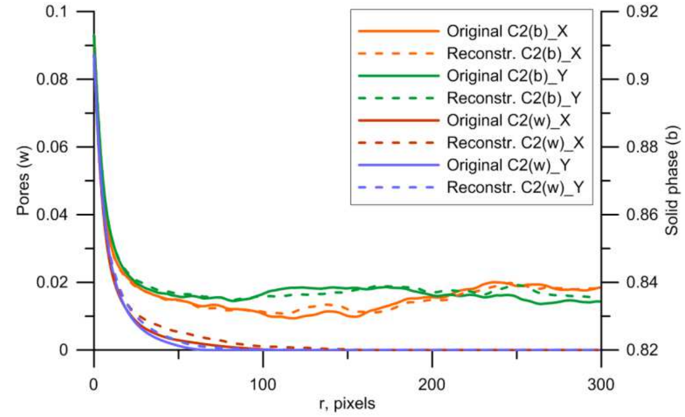

3

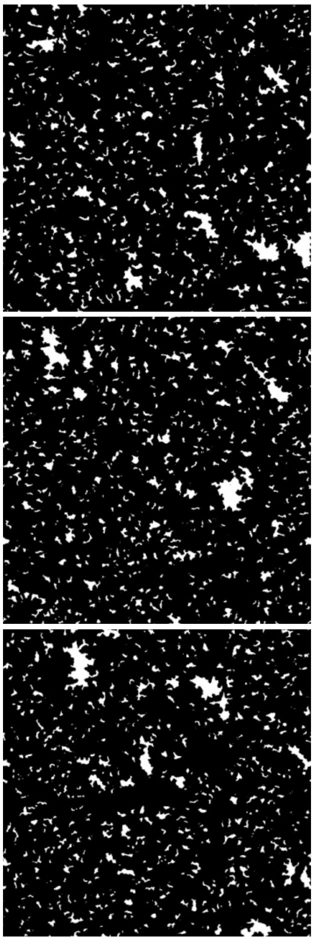

4

5

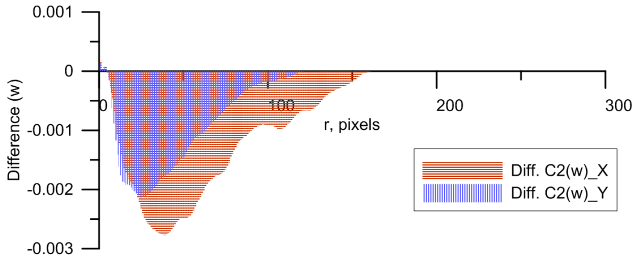

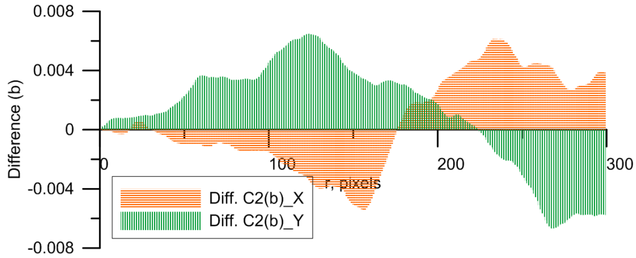

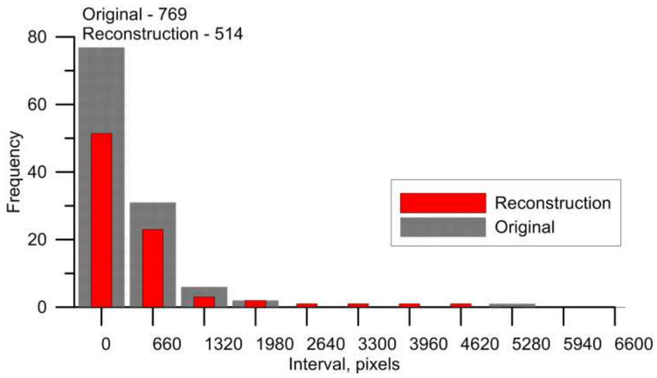

Sample	Shape					Orientation		
	1	2	3	4	5	6	7	8
Type I								
Original	0	2	11	32	55	30	32	38
Recon. 1	0	8	33	39	20	34	30	36
Recon. 2	0	6	35	39	20	33	27	40
Recon. 3*	0	7	35	36	22	31	30	39
Recon. 4	0	7	35	37	21	33	28	39
Recon. 5	0	7	34	39	20	32	27	41

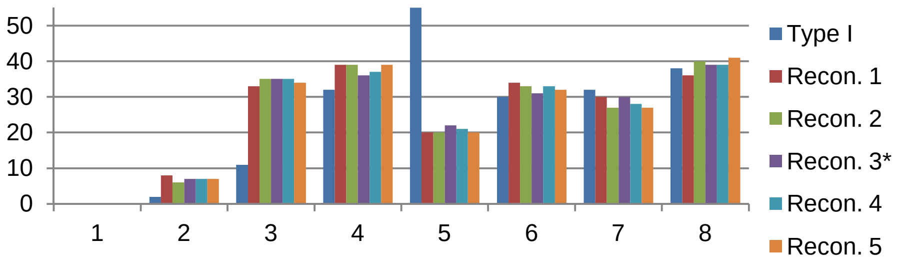

Type II

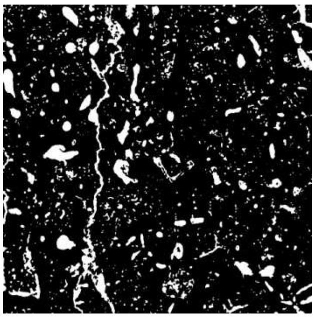

1

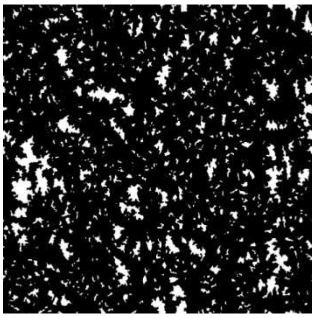

2

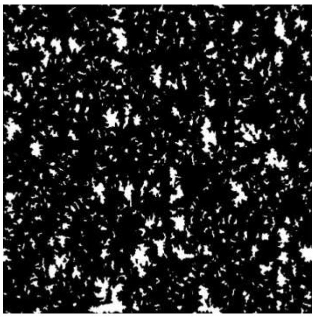

3

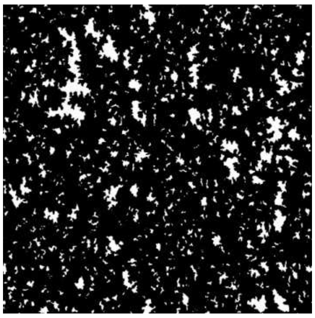

4

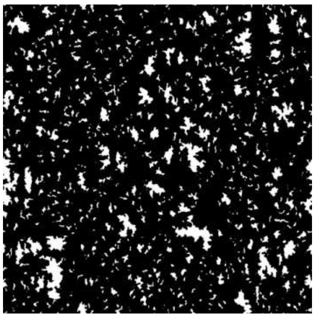

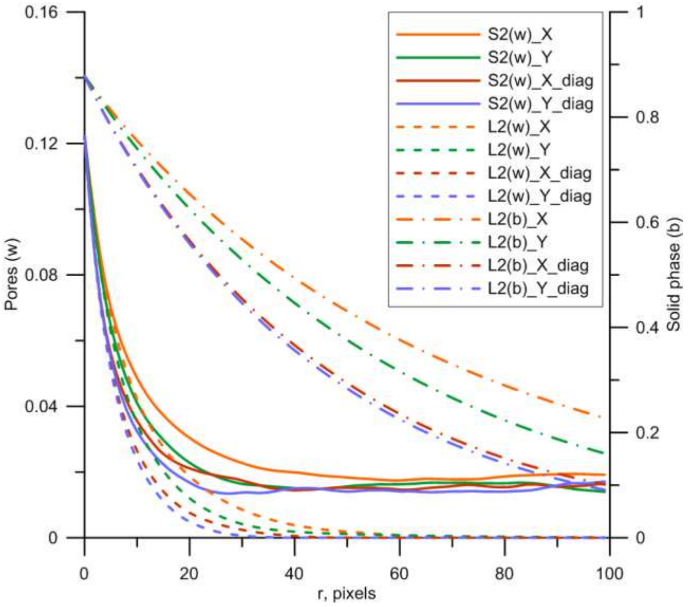
S2(w)_X
S2(w)_Y
S2(w)_X_diag
S2(w)_Y_diag
L2(w)_X
L2(w)_Y
L2(w)_X_diag
L2(w)_Y_diag
L2(b)_X
L2(b)_Y
L2(b)_X_diag
L2(b)_Y_diag
Pores (w)
Solid phase (b)
r, pixels

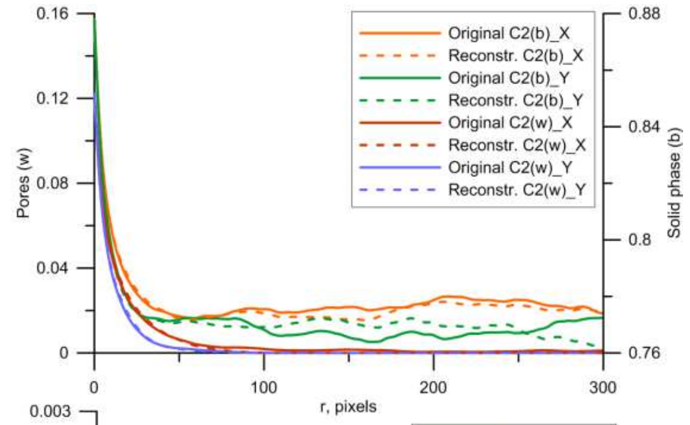
Original C2(b)_X
Reconstr. C2(b)_X
Original C2(b)_Y
Reconstr. C2(b)_Y
Original C2(w)_X
Reconstr. C2(w)_X
Original C2(w)_Y
Reconstr. C2(w)_Y
Pores (w)
Solid phase (b)
r, pixels

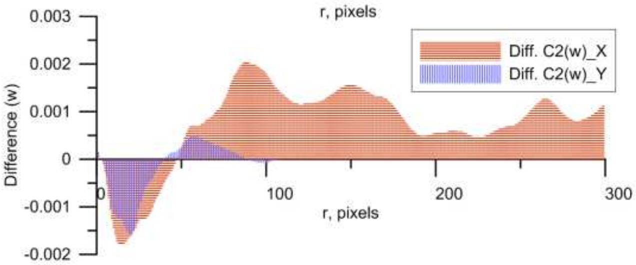
Diff. C2(w)_X
Diff. C2(w)_Y
Difference (w)
r, pixels

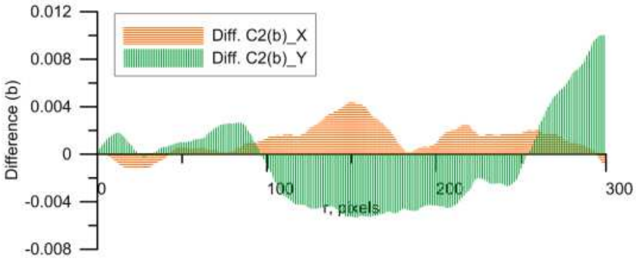
Diff. C2(b)_X
Diff. C2(b)_Y
Difference (b)
r, pixels

5

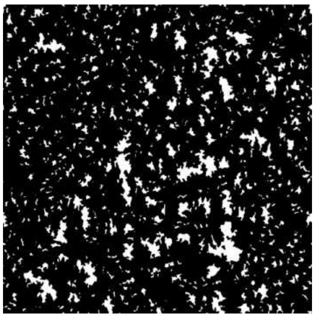

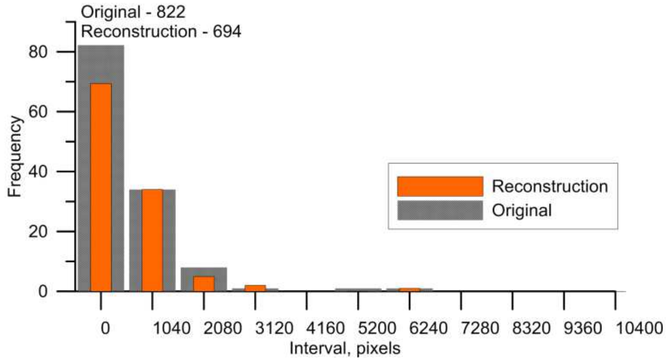

Sample	Shape					Orientation		
	1	2	3	4	5	6	7	8
Type II								
Original	0	3	16	38	43	42	29	29
Recon. 1*	0	7	30	41	22	44	27	29
Recon. 2	0	8	32	39	21	46	25	29
Recon. 3	0	7	30	41	22	45	27	28
Recon. 4	0	8	33	39	20	45	29	26
Recon. 5	0	7	30	42	21	47	27	26

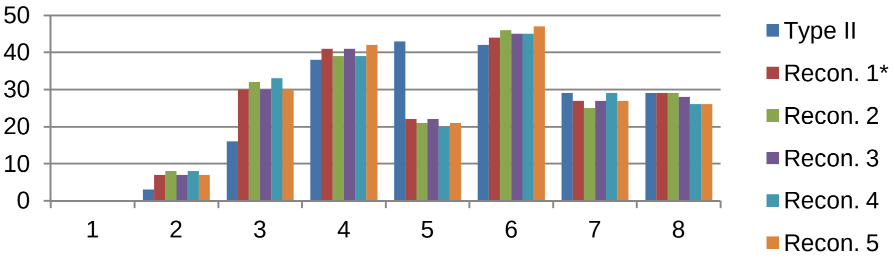

Type III

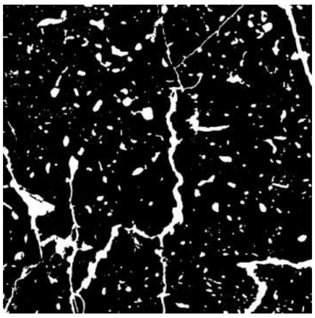

1 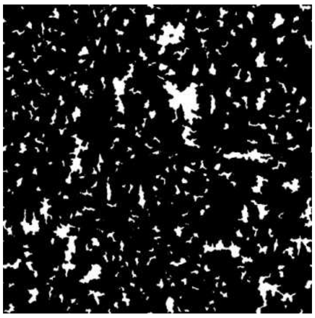

2 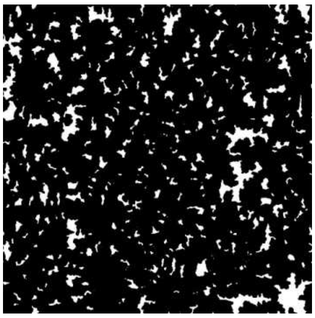

3 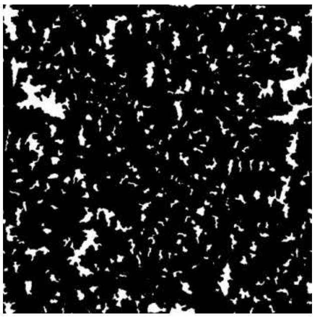

4 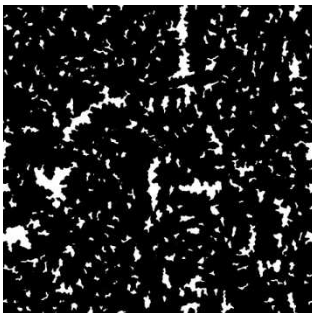

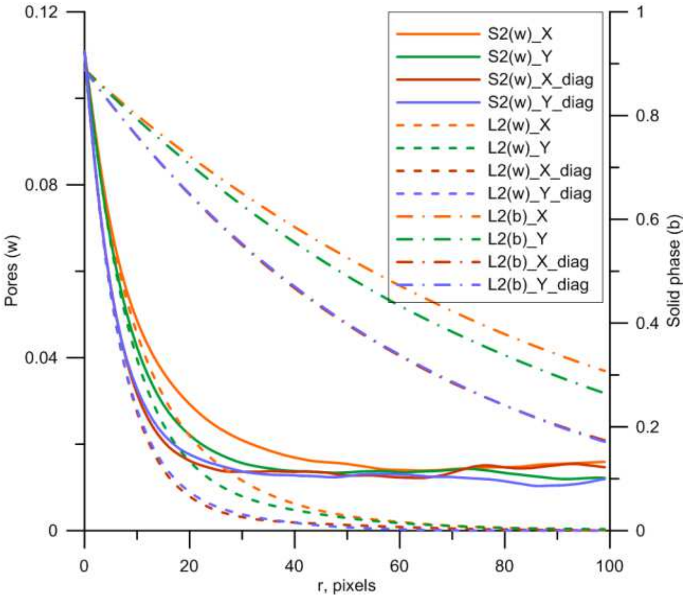

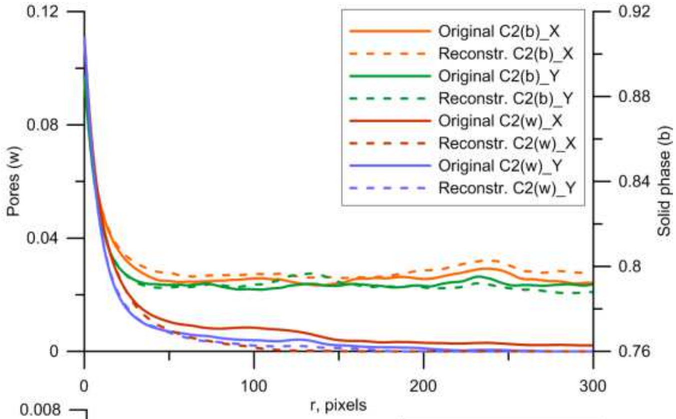

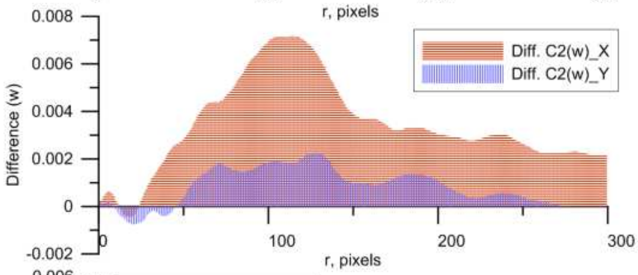

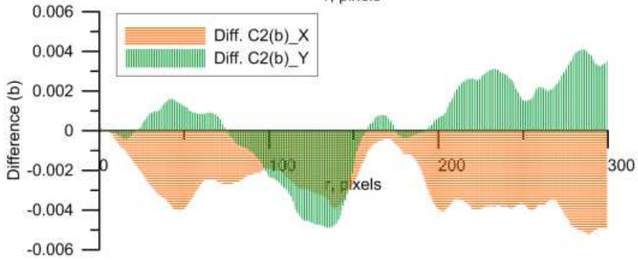

5

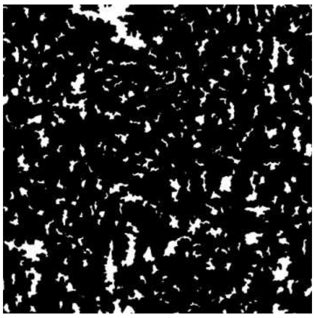

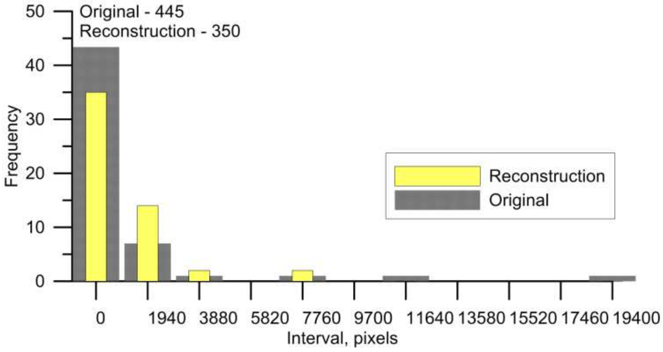

Sample	Shape					Orientation		
	1	2	3	4	5	6	7	8
Type III								
Original	1	5	14	35	45	29	33	38
Recon. 1	0	14	32	37	17	41	27	32
Recon. 2	0	12	37	35	16	40	28	32
Recon. 3*	0	12	32	37	19	41	29	30
Recon. 4	0	10	41	33	16	43	25	32
Recon. 5	0	16	33	35	16	44	26	30

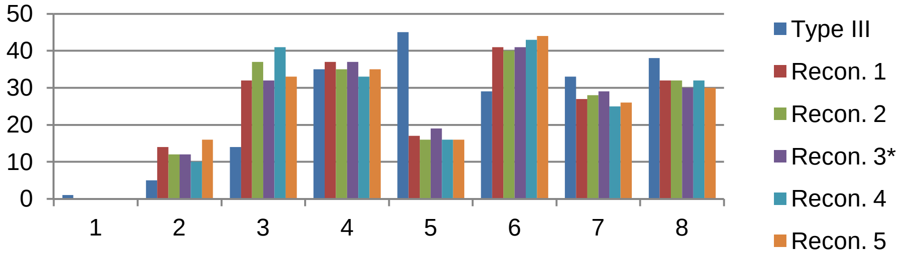

Type IV

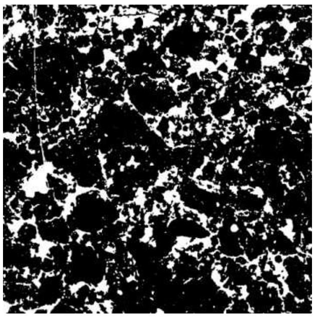

1

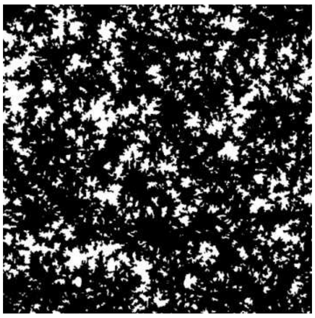

2

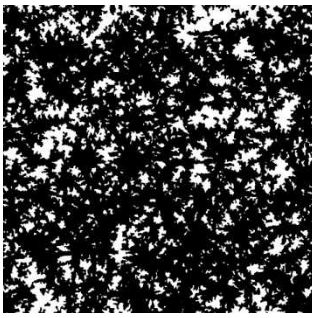

3

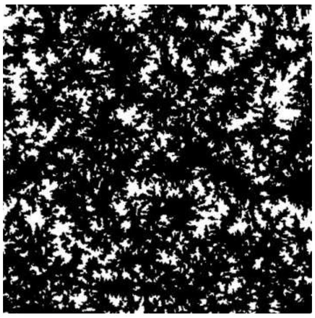

4

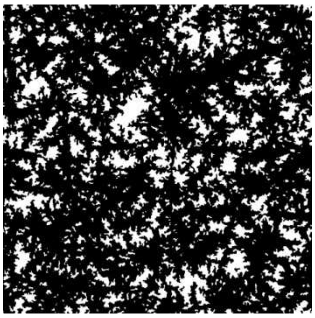

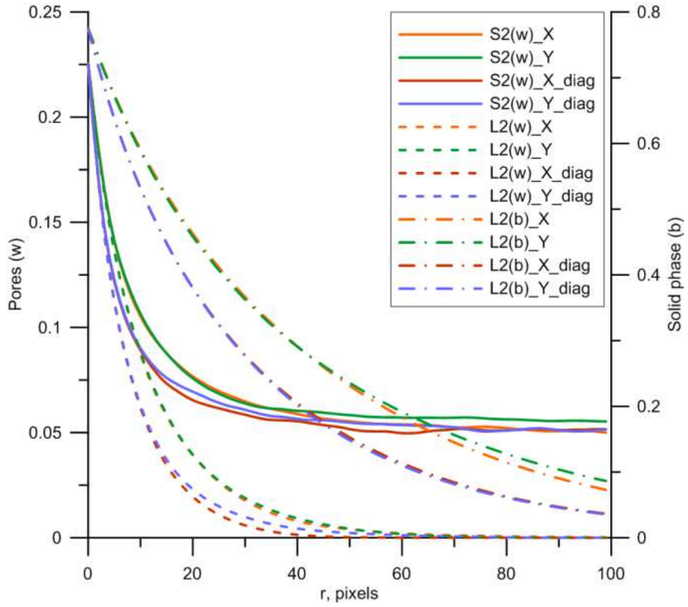
Pores (w)
Solid phase (b)
r, pixels
S2(w)_X
S2(w)_Y
S2(w)_X_diag
S2(w)_Y_diag
L2(w)_X
L2(w)_Y
L2(w)_X_diag
L2(w)_Y_diag
L2(b)_X
L2(b)_Y
L2(b)_X_diag
L2(b)_Y_diag

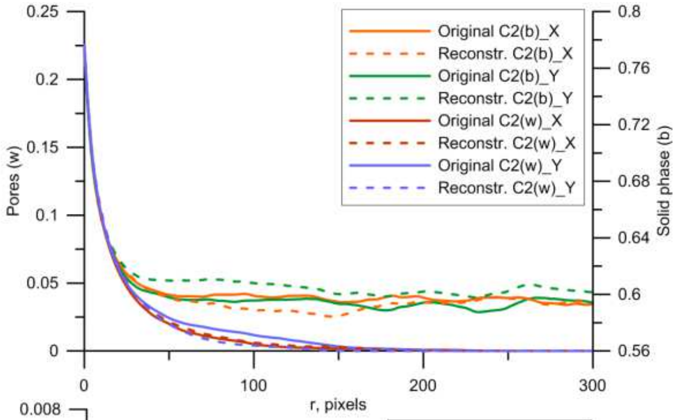
Pores (w)
Solid phase (b)
r, pixels
Original C2(b)_X
Reconstr. C2(b)_X
Original C2(b)_Y
Reconstr. C2(b)_Y
Original C2(w)_X
Reconstr. C2(w)_X
Original C2(w)_Y
Reconstr. C2(w)_Y

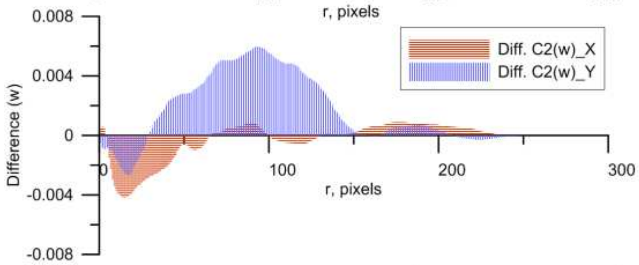
Difference (w)
r, pixels
Diff. C2(w)_X
Diff. C2(w)_Y

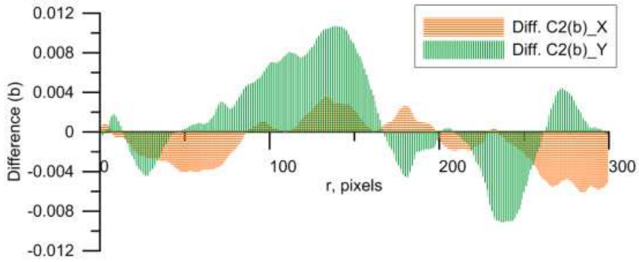
Difference (b)
r, pixels
Diff. C2(b)_X
Diff. C2(b)_Y

5

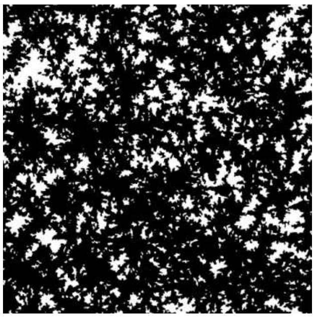

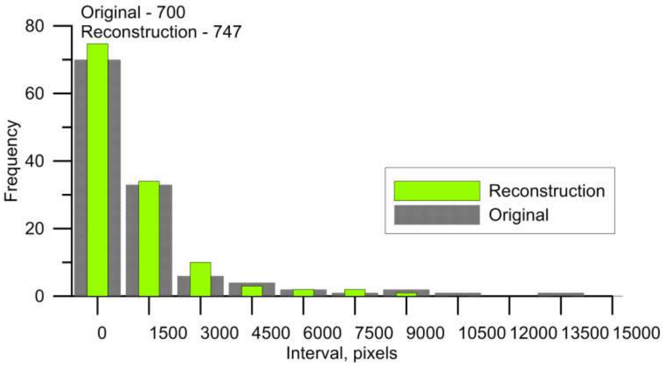

Sample	Shape					Orientation		
	1	2	3	4	5	6	7	8
Type IV								
Original	0	9	21	35	35	38	32	30
Recon. 1*	0	5	32	38	25	36	29	35
Recon. 2	0	6	34	40	20	39	25	36
Recon. 3	0	7	32	40	21	27	30	33
Recon. 4	0	7	31	39	23	36	27	37
Recon. 5	0	6	31	39	24	38	27	35

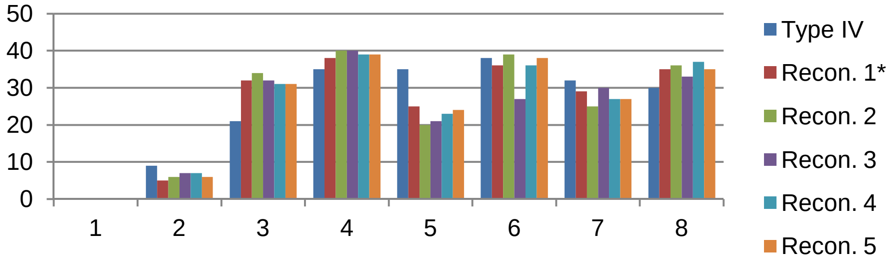

Type V

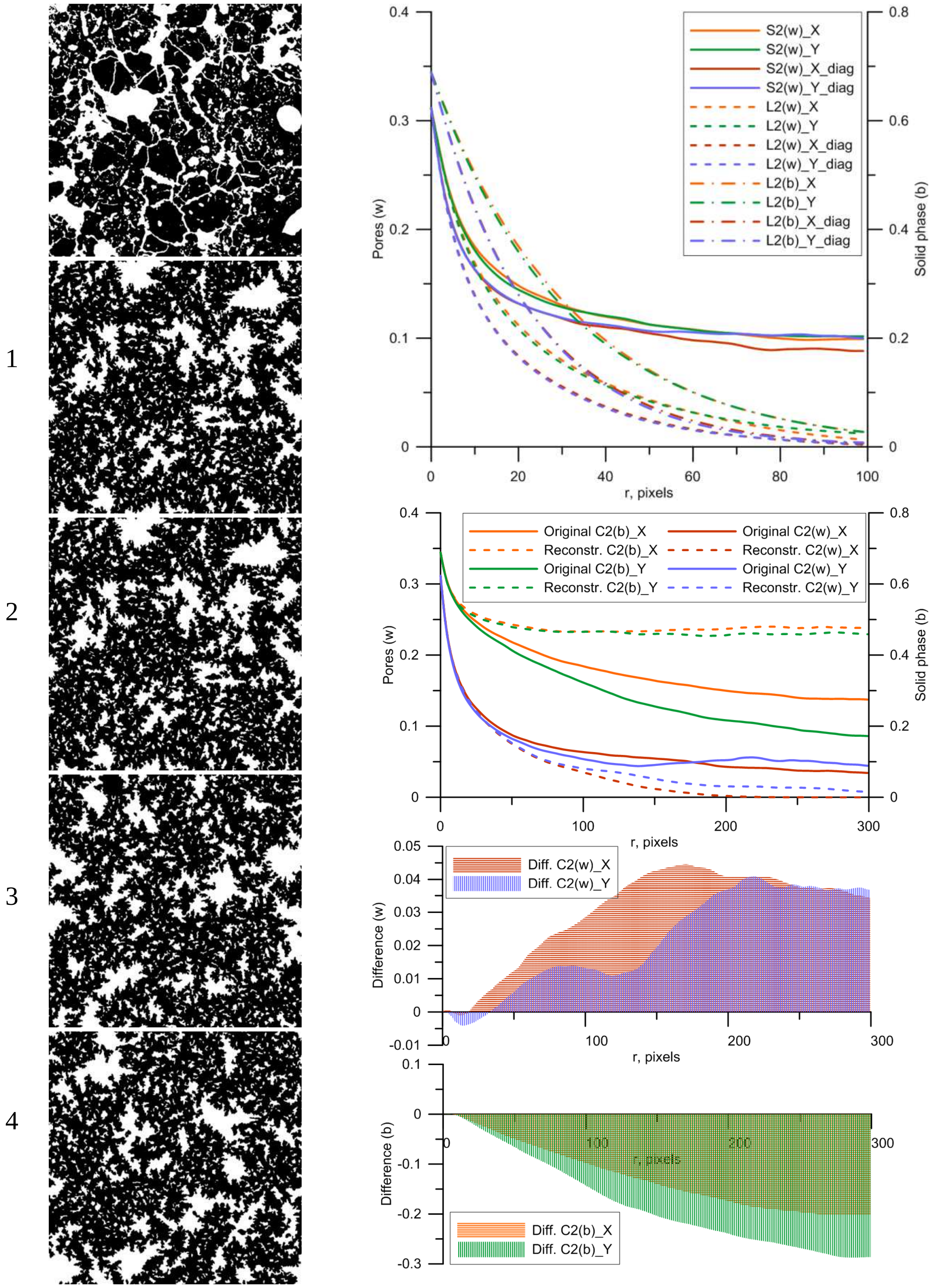
1
2
3
4
S2(w)_X
S2(w)_Y
S2(w)_X_diag
S2(w)_Y_diag
L2(w)_X
L2(w)_Y
L2(w)_X_diag
L2(w)_Y_diag
L2(b)_X
L2(b)_Y
L2(b)_X_diag
L2(b)_Y_diag
Pores (w)
Solid phase (b)
r, pixels
Original C2(b)_X
Reconstr. C2(b)_X
Original C2(b)_Y
Reconstr. C2(b)_Y
Original C2(w)_X
Reconstr. C2(w)_X
Original C2(w)_Y
Reconstr. C2(w)_Y
Diff. C2(w)_X
Diff. C2(w)_Y
Difference (w)
Diff. C2(b)_X
Diff. C2(b)_Y
Difference (b)

5

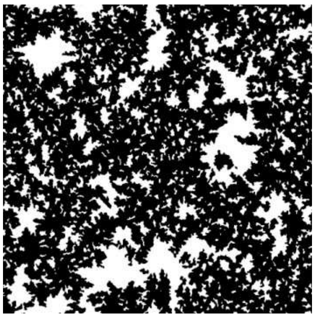

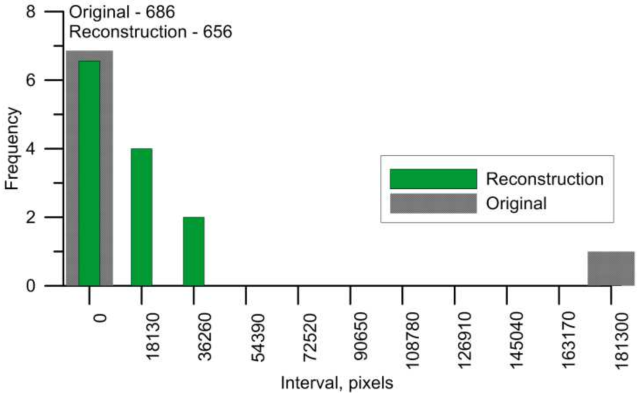

Sample	Shape					Orientation		
	1	2	3	4	5	6	7	8
Type V								
Original	0	9	17	39	35	39	29	32
Recon. 1	0	7	36	38	19	35	32	33
Recon. 2	0	10	35	38	17	37	30	33
Recon. 3*	0	9	30	40	21	36	33	31
Recon. 4	0	9	36	37	18	39	27	34
Recon. 5	0	8	34	40	18	40	28	32

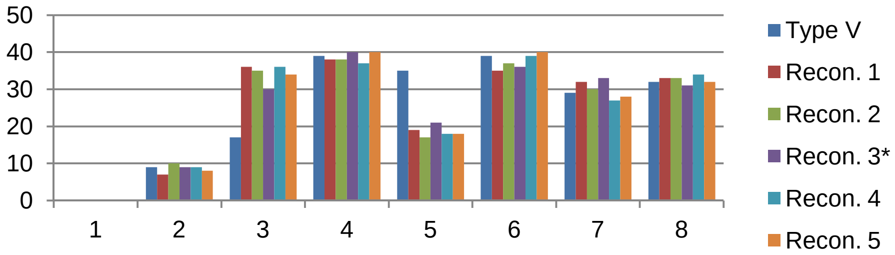

Type VI

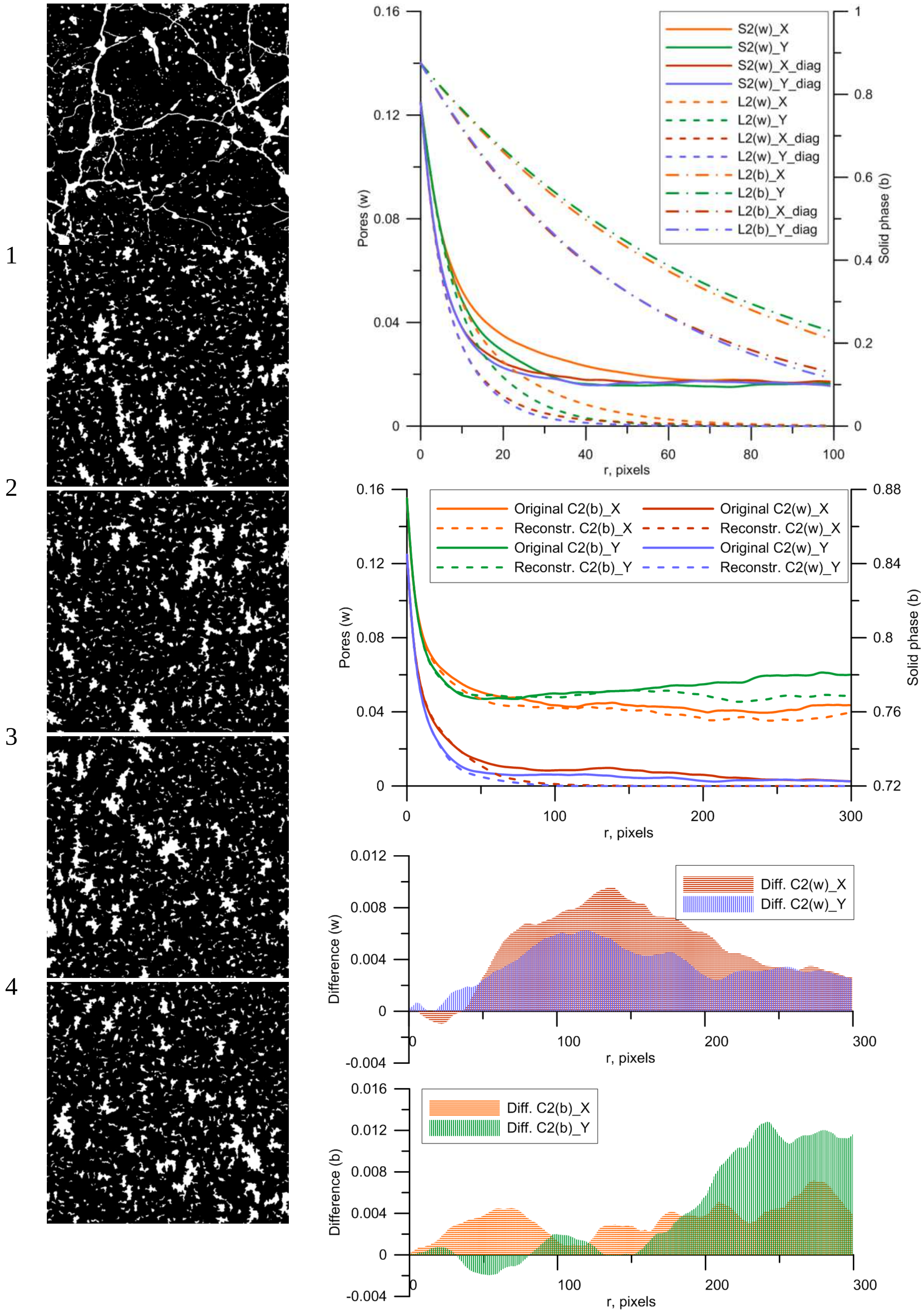
1
2
3
4
S2(w)_X
S2(w)_Y
S2(w)_X_diag
S2(w)_Y_diag
L2(w)_X
L2(w)_Y
L2(w)_X_diag
L2(w)_Y_diag
L2(b)_X
L2(b)_Y
L2(b)_X_diag
L2(b)_Y_diag
Pores (w)
Solid phase (b)
r, pixels
Original C2(b)_X
Reconstr. C2(b)_X
Original C2(b)_Y
Reconstr. C2(b)_Y
Original C2(w)_X
Reconstr. C2(w)_X
Original C2(w)_Y
Reconstr. C2(w)_Y
Pores (w)
Solid phase (b)
r, pixels
Diff. C2(w)_X
Diff. C2(w)_Y
Difference (w)
r, pixels
Diff. C2(b)_X
Diff. C2(b)_Y
Difference (b)
r, pixels

5

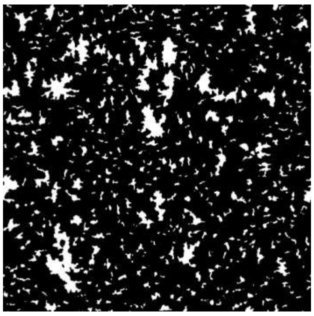

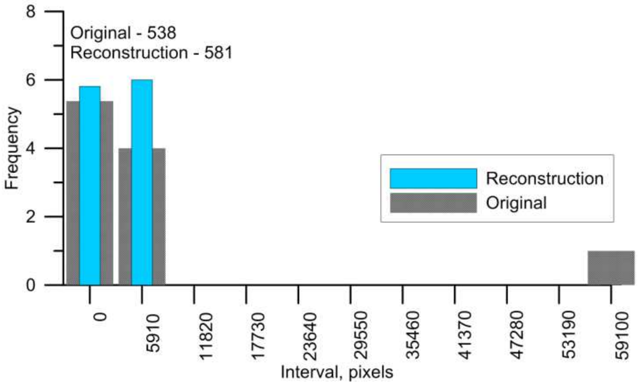

Sample	Shape					Orientation		
	1	2	3	4	5	6	7	8
Type VI								
Original	1	9	12	34	44	28	32	40
Recon 1	0	11	35	34	20	36	25	39
Recon 2	0	10	35	36	19	34	28	38
Recon 3	0	11	31	35	23	30	30	40
Recon 4	0	10	36	35	19	35	25	40
Recon 5	0	10	35	34	21	33	25	42

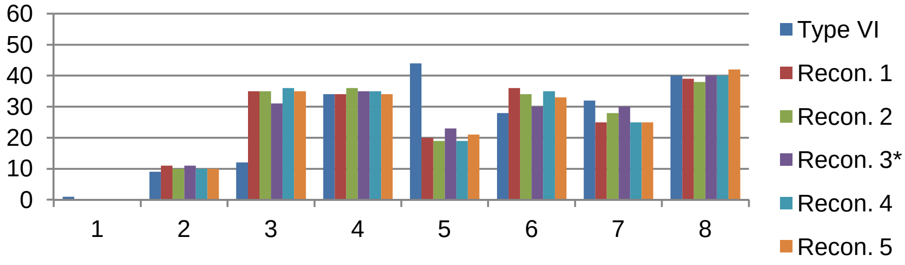

Type VII

1

2

3

4

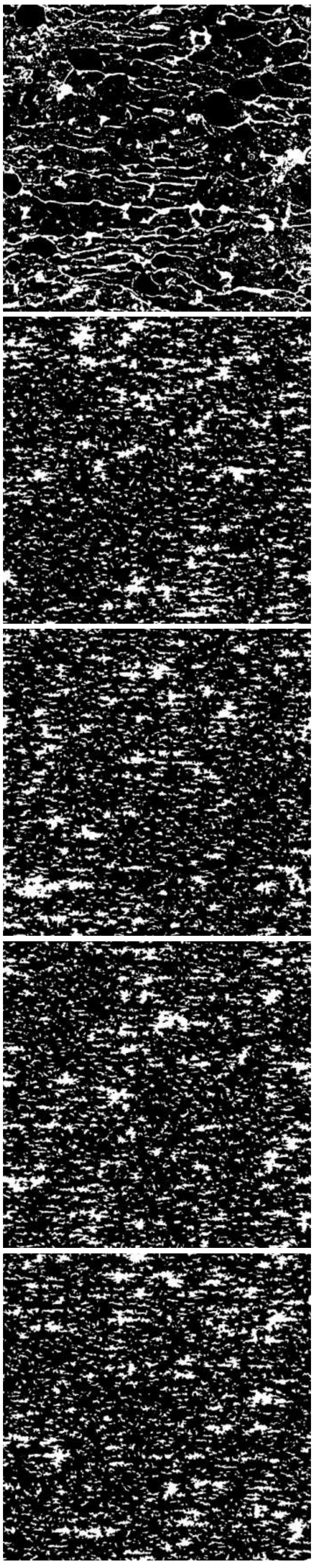

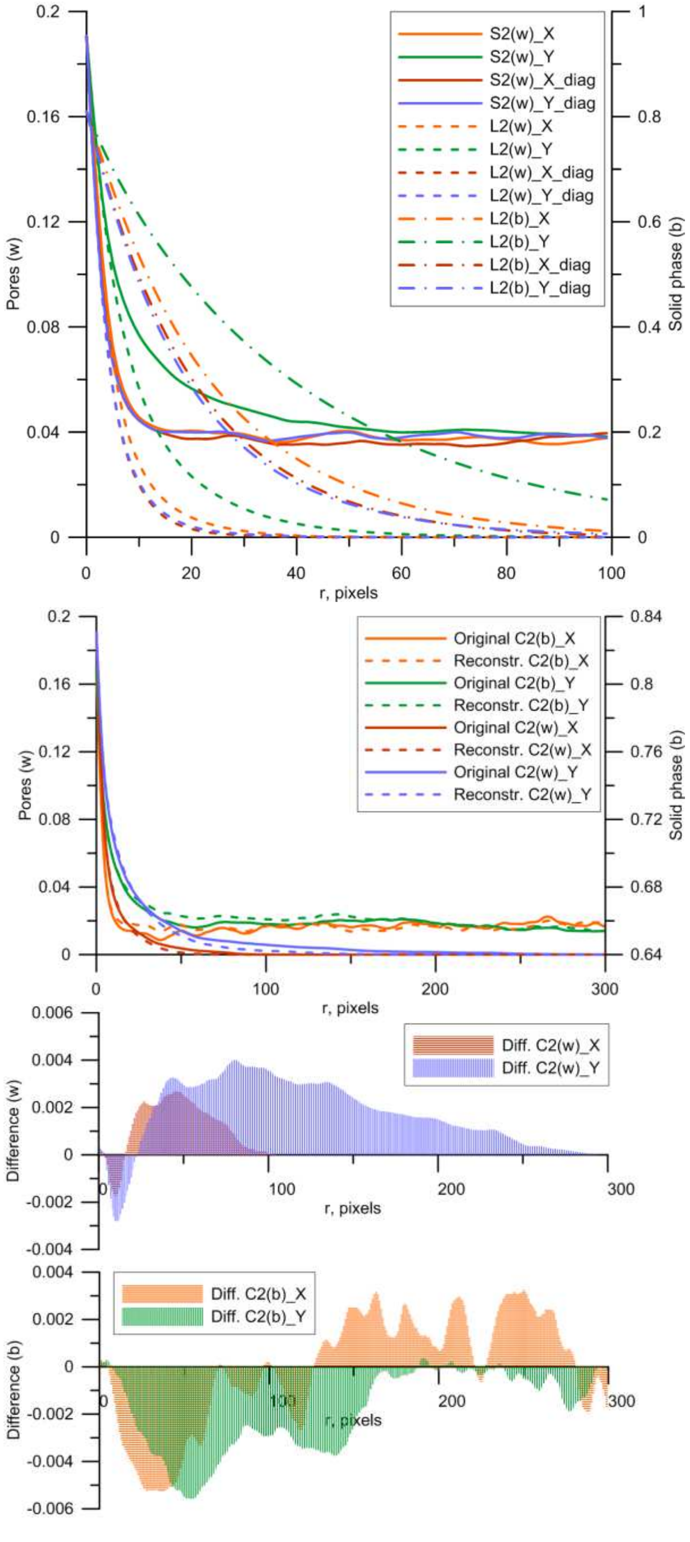

5

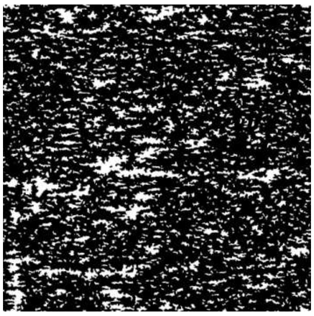

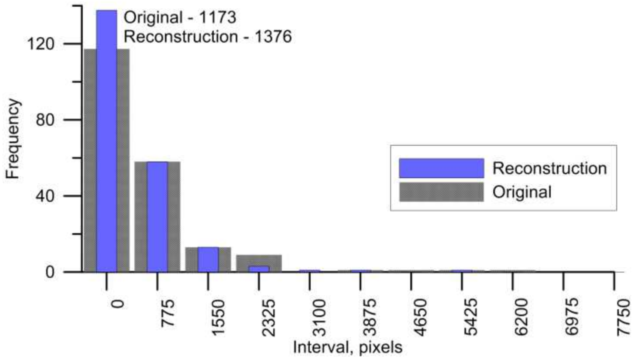

Sample	Shape					Orientation		
	1	2	3	4	5	6	7	8
Type VII								
Original	3	11	21	37	28	20	29	51
Recon. 1	0	13	30	35	22	16	21	63
Recon. 2	0	14	29	36	21	15	20	65
Recon. 3*	0	14	30	34	22	16	22	62
Recon. 4	0	12	30	36	22	16	21	63
Recon. 5	0	12	31	36	21	15	20	65

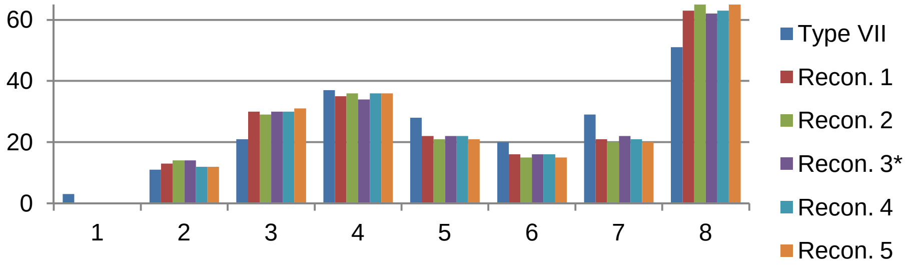

Type VIII

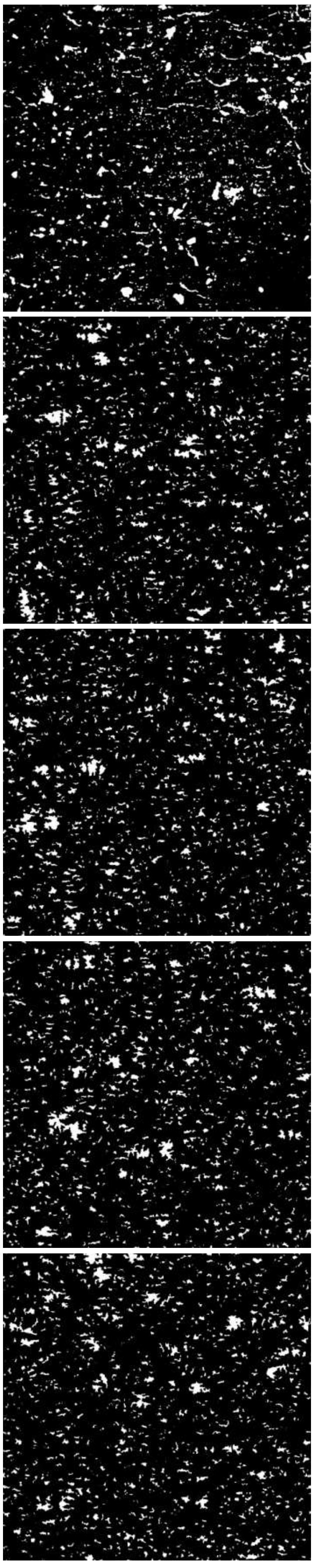

1

2

3

4

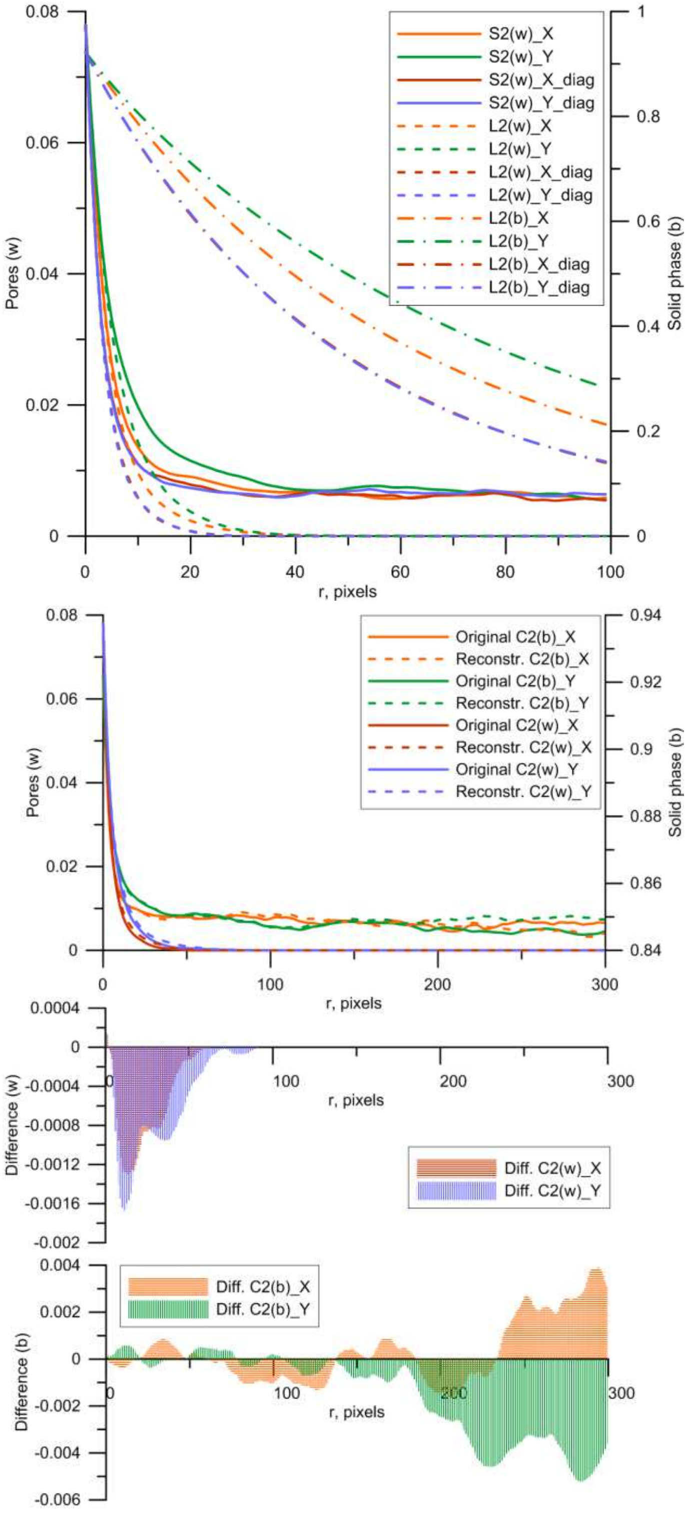

5

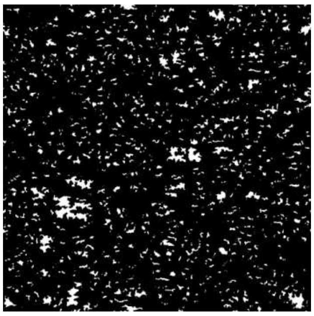

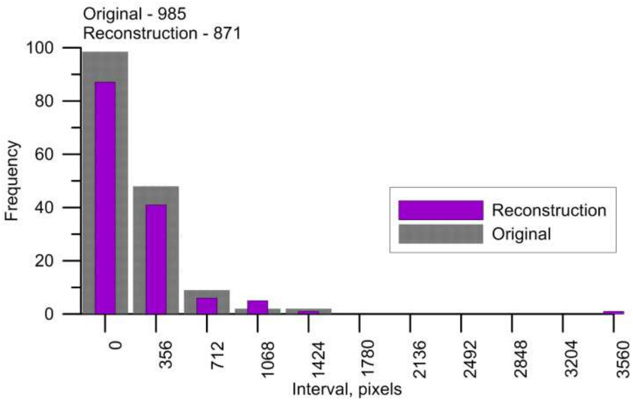

Sample	Shape					Orientation		
	1	2	3	4	5	6	7	8
Type VIII								
Original	0	5	12	40	43	25	28	47
Recon. 1	0	5	27	41	27	27	25	48
Recon. 2	0	6	26	42	26	26	26	48
Recon. 3	0	5	30	45	20	22	27	51
Recon. 4	0	6	26	42	26	25	26	49
Recon. 5*	0	6	28	38	28	26	26	48

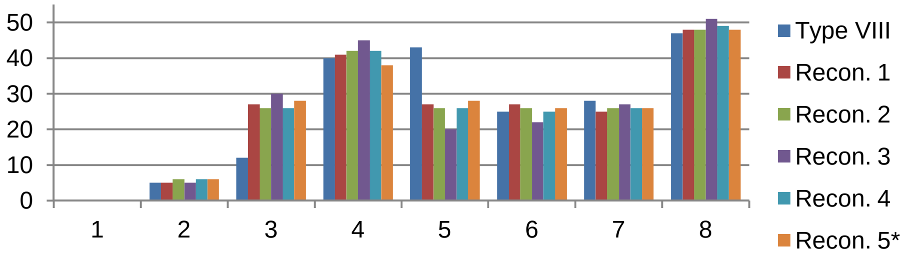

Supplement: S1 File — In this material for each of eight soil types we report: 1) original image of the thin-section, 2) all five reconstructions obtained using the method described in paper, 3) a set of S 2 -L 2 correlation functions for original image (all reconstructions have similar correlation functions up to a tolerance of E = 10–7, all minor differences would be invisible on the such a graph), 4) a comparison of cluster functions computed for original image and best replica judging by differences in cluster function values, 5) comparison of pore-size distributions for original thin-section and the best replica judging by morphology analysis, 6) a table with results of morphological analysis covering all replicas and the original. (PDF) [file pone.0126515.s001.pdf]
